# *Clostridioides difficile* Infection in a Romanian Tertiary Care Hospital: Epidemiology, Comorbidity Burden, Treatment Patterns, and Mortality Outcomes (2023–2025)

**DOI:** 10.3390/antibiotics15101021

**Published:** 2026-10-14

**Authors:** Tania Vlad, Daliborca Cristina Vlad, Calin Muntean, Corina Flangea, Victor Dumitrașcu, Roxana Popescu, Mihai Mitulețu, Cristian Sebastian Vlad

**Affiliations:** 1 Doctoral School, Faculty of Medicine, “Victor Babeș” University of Medicine and Pharmacy, 2 Eftimie Murgu Square, 300041 Timisoara, Romania; tania.vlad@umft.ro (T.V.); vlad.cristian@umft.ro (C.S.V.); 2 ANAPATMOL Research Center, Department of Cell and Molecular Biology, Faculty of Medicine, “Victor Babeș” University of Medicine and Pharmacy, 2 Eftimie Murgu Square, 300041 Timisoara, Romania; popescu.roxana@umft.ro; 3 Department of Biochemistry and Pharmacology, Faculty of Medicine, “Victor Babeș” University of Medicine and Pharmacy, 2 Eftimie Murgu Square, 300041 Timisoara, Romania; flangea.corina@umft.ro (C.F.); dumitrascu.victor@umft.ro (V.D.); 4 Clinical Laboratory Department, “Pius Brinzeu” County Emergency Hospital, 156 Liviu Rebreanu Blvd, 300723 Timisoara, Romania; mihai.mituletu@umft.ro; 5 Department of Functional Sciences, Medical Informatics and Biostatistics, Faculty of Medicine, “Victor Babeș” University of Medicine and Pharmacy, 2 Eftimie Murgu Square, 300041 Timisoara, Romania

**Keywords:** *Clostridioides difficile*, healthcare-associated infection, in-hospital mortality, comorbidity, sepsis, antimicrobial stewardship, Romania, retrospective cohort, rule-based information extraction, electronic health records

## Abstract

**Background:** Real-world outcome data on *Clostridioides difficile* infection (CDI) from Eastern Europe are scarce, and partly digitised hospital records make surveillance laborious. We characterised the epidemiology, comorbidity, treatment and in-hospital mortality of CDI in a Romanian tertiary hospital and validated a rule-based pipeline for extracting surveillance variables from free-text records. **Methods:** Retrospective two-period cohort (1 September 2023 to 31 December 2025) of 295 CDI episodes at the Pius Brinzeu County Emergency Clinical Hospital, Timisoara. Thirty variables were extracted from narrative records with a lexical algorithm applied identically to both periods and validated against manual coding (*n* = 144). Mortality predictors were analysed by logistic regression with LASSO selection, bootstrap-corrected discrimination and calibration testing. **Results:** Mean age was 69.2 years. Hypertension (76.6%), heart failure (46.4%), neoplasm (39.3%), ascites or pleural effusion (61.7%), sepsis (56.9%) and pneumonia (44.4%) were common; vancomycin was documented in 83.7% of episodes and non-CDI antibiotics in 85.8%. In-hospital mortality during the index admission was 33.6%. Sepsis (adjusted OR 2.68), pneumonia (2.53) and ascites or pleural effusion (2.32) independently predicted death (optimism-corrected AUC 0.75), whereas individual chronic comorbidities lost significance after adjustment; mortality rose from 6.8% with none of the three acute markers to 61.1% with all three. Toxin index magnitude was unrelated to death. The second period had a higher comorbidity score (3.1 vs. 2.5, *p* = 0.002). Extraction reached at least 90% accuracy for 15 of 22 variables (median kappa 0.89). **Conclusions:** Acute organ involvement, much more than underlying comorbidity, determines in-hospital CDI mortality in this setting. Rule-based extraction supports reproducible CDI surveillance in partially digitised hospitals when low-agreement variables are verified by hand.

## 1. Introduction

*Clostridioides difficile* is a spore-forming, toxin-producing anaerobe and the most frequent identifiable cause of healthcare-associated diarrhoea in high-income countries [1,2]. Disease results from the action of toxins A and B on the colonic epithelium and ranges from self-limiting diarrhoea to pseudomembranous colitis, toxic megacolon, perforation and death [3]. Some strains, including the epidemic PCR-ribotype 027 lineage, also produce the binary toxin CDT, which disrupts the actin cytoskeleton of enterocytes, increases bacterial adherence and has been linked to more severe disease and higher case fatality [4,5]. In the United States, an estimated 462,000 cases occurred in 2017, and although healthcare-associated incidence has since declined, community-associated infection has not [6]. European point-prevalence work has shown that *C. difficile* remains among the leading pathogens of healthcare-associated infection in acute-care hospitals [7], that a substantial proportion of cases go undiagnosed because the test is not requested [8], and that outcomes vary widely between countries [9]. Systematic reviews of the global and European literature place the pooled incidence of healthcare-associated CDI at about 3.5 cases per 10,000 patient-days, with recurrence in a substantial minority of patients and a considerable excess in length of stay and cost [10,11,12,13]. Surveillance-based incidence reports from thirteen countries, and burden reviews from China, Argentina and Italy, confirm that the problem is global and that data quality differs markedly between regions [14,15,16,17]. The worldwide spread of the epidemic PCR-ribotype 027 lineage in the early 2000s changed the clinical picture towards more severe disease in older patients [18,19].

Host factors shape both the risk of acquiring CDI and the risk of dying from it. Prior antibiotic exposure is the dominant modifiable risk factor, with clindamycin, cephalosporins, carbapenems and fluoroquinolones carrying the highest pooled odds [20]. Advanced age, frailty and cognitive impairment interact with the infection in a bidirectional way [21,22]. Systematic reviews of hospital-acquired CDI list comorbidity burden, immunosuppression, prior hospitalisation and acid suppression among the consistent predictors [23,24,25]. Specific chronic conditions have been linked to worse outcomes: chronic obstructive pulmonary disease [26], chronic and end-stage kidney disease [27], diabetes mellitus [28,29], active cancer [30] and non-alcoholic fatty liver disease [31]. Mortality after CDI is driven by age, leucocytosis, hypoalbuminaemia, renal impairment and concomitant systemic antibiotics, as summarised in two systematic reviews [32,33] and as operationalised in bedside scores such as ATLAS and CARDS [34,35]. A Dutch multicentre cohort showed that a large part of the deaths after CDI are attributable to the infection itself rather than to patients’ underlying comorbidities [36]. Newer biomarkers and quantitative toxin measurements have been proposed for early severity prediction, with inconsistent results so far [37,38,39].

Treatment recommendations have changed over the last decade. The European Society of Clinical Microbiology and Infectious Diseases (ESCMID) 2021 guidance and the 2021 IDSA/SHEA focused update both prefer fidaxomicin for an initial episode, retain oral vancomycin as an acceptable alternative, and restrict metronidazole to settings where neither agent is available [40,41,42,43]. The shift rests on randomised evidence that fidaxomicin reduces recurrence compared with vancomycin [44] and that metronidazole is inferior to vancomycin, particularly in severe disease [45,46]. Concomitant antibiotics for other infections lower cure rates and raise recurrence [47], and the evidence supporting probiotics for prevention is of low certainty [48]. Real-world series from referral centres show that adherence to these recommendations is incomplete, even in well-resourced settings [49].

Data from Romania remain limited. Ribotype 027 became dominant in Romanian hospitals in the early 2010s [50,51], CDI is now the healthcare-associated infection most often reported to national surveillance [52,53], and antibiotic consumption is among the highest in the European Union [54]. Outcome data from large tertiary hospitals are nonetheless scarce, partly because clinical records are often kept as narrative documents, from which surveillance variables must be abstracted by hand or with information-extraction tools that have rarely been evaluated on Romanian-language text [55,56,57,58,59,60].

We therefore had two objectives. The first was to describe the epidemiology, comorbidity burden, treatment patterns and in-hospital mortality of laboratory-confirmed CDI over two consecutive periods in a large tertiary hospital in western Romania, and to identify independent predictors of death. The second was to build and validate a pragmatic, fully reproducible, rule-based pipeline that converts unstructured Romanian clinical records into a structured CDI surveillance dataset, and to quantify where such a pipeline can be trusted and where it cannot.

## 2. Results

### 2.1. Laboratory Testing Context

**Figure 1:**
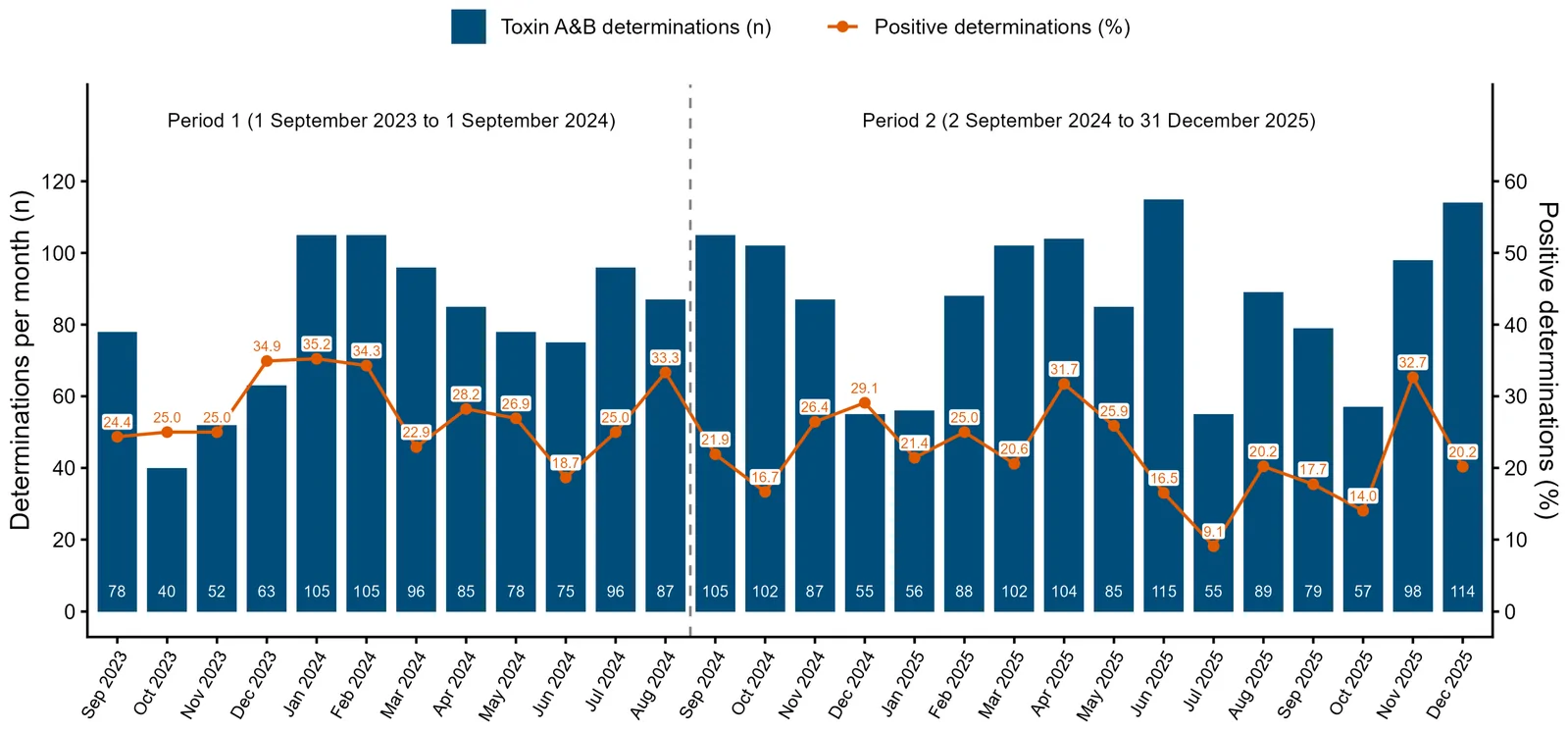
Monthly number of *C. difficile* toxin A&B determinations (bars, left axis; counts at the base of each bar) and proportion of positive determinations (index ≥ 1.0; line, right axis; percentage above each point), September 2023 to December 2025. The dashed line separates Period 1 (1 September 2023 to 1 September 2024) from Period 2 (2 September 2024 to 31 December 2025); the September 2024 bar includes the single determination performed on 1 September 2024.

Between 2 September 2023 and 30 December 2025 the hospital laboratory performed 2351 *C. difficile* toxin A&B determinations: 961 in the first period (12 months, 80.1 per month) and 1390 in the second (16 months, 86.9 per month). Using the laboratory positivity threshold (index ≥ 1.0), 271 determinations (28.2%) were positive in the first period and 308 (22.2%) in the second, giving an overall per-determination positivity of 24.6% (579/2351). Monthly volume ranged from 40 (October 2023) to 115 (June 2025) determinations, and monthly positivity from 9.1% (July 2025) to 35.2% (January 2024), with the highest positivity clustered in the winter months of December 2023 to February 2024 (Figure 1). Tested individuals had a mean age of 64.7 years and 49.8% were male. Positivity increased with age: 12.6% (11/87) in patients under 18 years, 21.2% (161/759) at 18 to 64 years, 25.4% (272/1072) at 65 to 79 years and 31.2% (135/433) at 80 years or older (*p* < 0.001 for the comparison of age between positive and negative determinations).

### 2.2. Cohort Characteristics

A total of 295 CDI episodes with an available clinical record were analysed (Period 1, *n* = 145; Period 2, *n* = 150). In 293 episodes the maximum toxin A&B index was at or above the positivity threshold; two records with a sub-threshold index were retained because CDI was explicitly diagnosed and documented in the clinical narrative. A single toxin determination was performed in 204 patients (69.2%), two in 60 (20.3%), three in 21 (7.1%) and four to six in 10 (3.4%). Mean age was 69.2 ± 14.8 years (median 72, range 5 to 95); 80 patients (27.1%) were younger than 65 years, 147 (49.8%) were 65 to 79 years and 68 (23.1%) were 80 years or older. Three patients were younger than 18 years (aged 5, 15 and 15; two in Period 1 and one in Period 2; all female); none of them died. They are shown as a separate age group in Table 1; the 292 adults had a mean age of 69.8 ± 13.7 years and an in-hospital mortality of 33.9% (99/292). Women slightly outnumbered men (151, 51.2% vs. 144, 48.8%). Two-thirds of patients resided in Timis County (200, 67.8%); the remainder came mainly from the neighbouring counties of Caras-Severin (28, 9.5%), Arad (20, 6.8%), Hunedoara (20, 6.8%), Gorj (10, 3.4%) and Mehedinti (10, 3.4%), reflecting the hospital’s regional referral function.

The comorbidity burden was heavy (Table 1). Hypertension was documented in 226 patients (76.6%), heart failure in 137 (46.4%), neoplasm in 116 (39.3%), diabetes mellitus in 115 (39.0%), chronic kidney disease in 91 (30.8%), prior stroke in 84 (28.5%) and chronic obstructive pulmonary disease or asthma in 61 (20.7%). The unweighted comorbidity score (number of these seven conditions, 0 to 7) had a mean of 2.8 ± 1.7 and a median of 3; only 24 patients (8.1%) had none of the seven conditions, whereas 99 (33.6%) had four or more. The age-adjusted partial Charlson Comorbidity Index (CCI), computed from the six Charlson conditions captured by the extraction (Section 4.4), had a mean of 5.3 ± 2.6 (median 5, interquartile range 4–7; 2.7 ± 2.1 without age points) and correlated closely with the unweighted score (Spearman rho 0.92). Acute conditions accompanying the CDI episode were also frequent: ascites or pleural effusion in 182 patients (61.7%), sepsis or septic shock in 168 (56.9%), pneumonia in 131 (44.4%), urinary tract infection in 105 (35.6%) and gastrointestinal bleeding in 52 (17.6%). When sepsis, pneumonia and ascites/pleural effusion were counted together as acute-severity markers, 44 patients (14.9%) had none, 93 (31.5%) had one, 86 (29.2%) had two and 72 (24.4%) had all three. Computed tomography was performed in 235 patients (79.7%) and endoscopy in 78 (26.4%).

Intensive care management was required in 134 patients (45.4%), and transfer to the regional infectious diseases hospital was documented in 152 (51.5%). In-hospital death during the index admission occurred in 99 patients (33.6%). Mortality was 61.2% (82/134) among patients managed in intensive care and 10.6% (17/161) among the others.

### 2.3. Trends Between Periods

**Figure 2:**
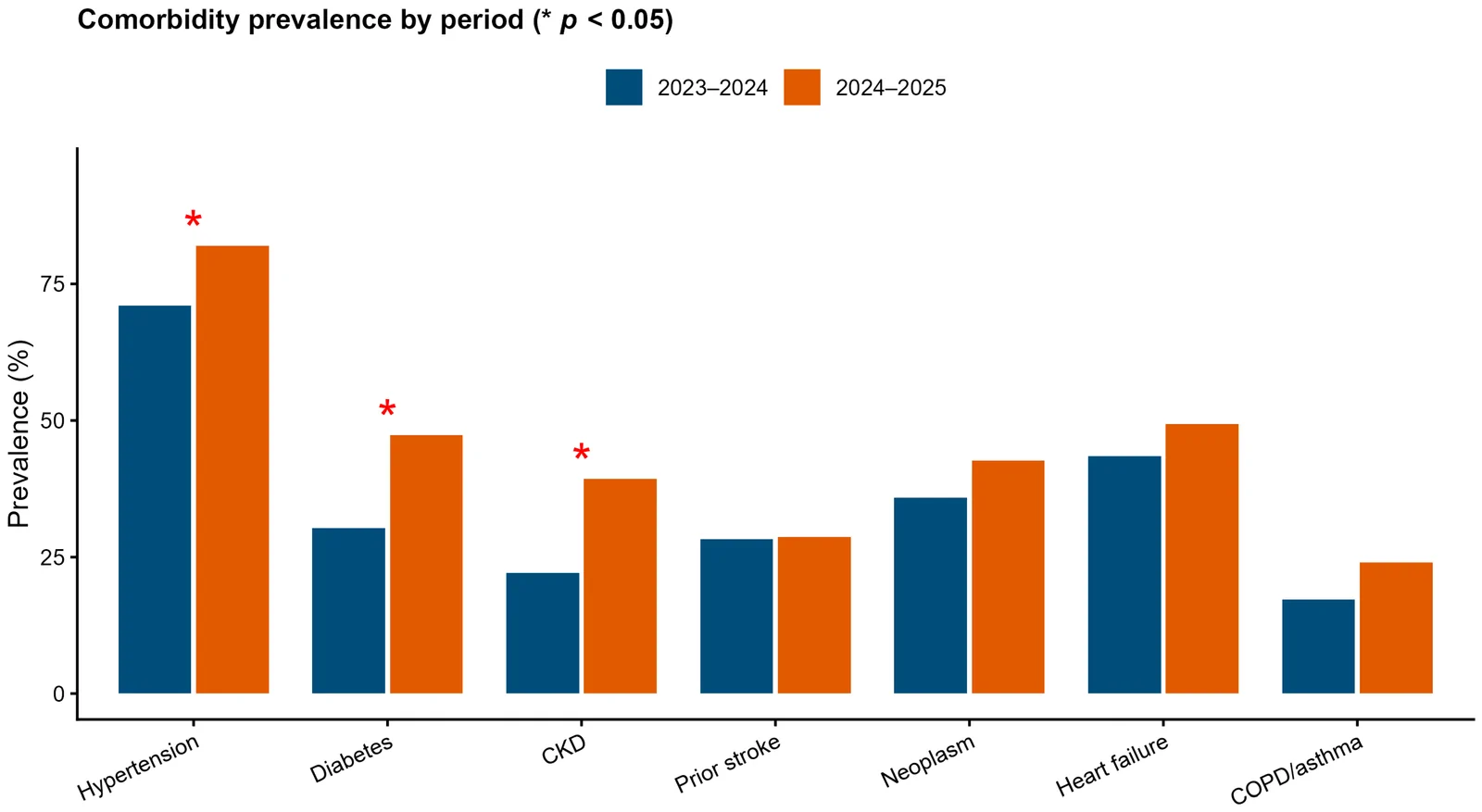
Prevalence of the seven chronic comorbidities in the two study periods (* *p* < 0.05, chi-squared test). Blue bars, Period 1 (2023–2024); orange bars, Period 2 (2024–2025). CKD, chronic kidney disease; COPD, chronic obstructive pulmonary disease.

Because the two periods differed in length (12 and 16 months), the number of episodes is not a measure of incidence: crudely normalised, it corresponds to 12.1 episodes per month in Period 1 and 9.4 per month in Period 2, and patient-day denominators, which would allow incidence rates per 10,000 patient-days, were not available. Age and sex distribution did not differ between periods (Table 1). Patients in the second period carried a higher comorbidity score (mean 3.1 ± 1.8 vs. 2.5 ± 1.5; median 3 vs. 2; *p* = 0.002), and the proportion with four or more chronic conditions rose from 24.1% (35/145) to 42.7% (64/150); the age-adjusted partial CCI rose accordingly (mean 5.7 ± 2.7 vs. 4.8 ± 2.4, *p* = 0.004). Three individual comorbidities were significantly more prevalent in the second period: hypertension (82.0% vs. 71.0%, *p* = 0.026), diabetes mellitus (47.3% vs. 30.3%, *p* = 0.003) and chronic kidney disease (39.3% vs. 22.1%, *p* = 0.001) (Figure 2). Gastrointestinal bleeding was documented twice as often (23.3% vs. 11.7%, *p* = 0.009), and endoscopy was performed almost twice as often (34.7% vs. 17.9%, *p* = 0.001), whereas computed tomography use fell slightly (75.3% vs. 84.1%, *p* = 0.060). The frequency of sepsis (61.3% vs. 52.4%), pneumonia (42.0% vs. 46.9%) and ascites/pleural effusion (60.0% vs. 63.4%) did not differ, and the mean number of acute-severity markers was identical (1.6 in both periods). Intensive care requirement (49.3% vs. 41.4%, *p* = 0.170), transfer (54.7% vs. 48.3%, *p* = 0.272) and in-hospital mortality (36.0% vs. 31.0%, *p* = 0.367) were numerically but not significantly higher in the second period. The maximum toxin A&B index was lower in the second period (median 28.7 vs. 41.6; *p* = 0.091). These comparisons were not prespecified and involve 30 tests. After Benjamini–Hochberg correction, the differences in comorbidity score, partial CCI, diabetes, chronic kidney disease and endoscopy (q ≤ 0.021) and in gastrointestinal bleeding (q = 0.044) remained below 0.05, whereas those in hypertension (q = 0.112) and in the distribution of treatment regimens (q = 0.135) did not; with the more conservative Holm procedure, only chronic kidney disease (adjusted *p* = 0.039) and endoscopy (adjusted *p* = 0.034) remained significant. The between-period differences should therefore be read as exploratory.

### 2.4. Treatment Patterns

Vancomycin was documented in 247 episodes (83.7%) and metronidazole in 97 (32.9%). Vancomycin alone was the most frequent regimen (174, 59.0%), followed by vancomycin combined with metronidazole (73, 24.7%), metronidazole alone (24, 8.1%) and no documented CDI-specific agent (24, 8.1%) (Table 2). The distribution shifted between periods (*p* = 0.036): metronidazole monotherapy fell from 12.4% (18/145) to 4.0% (6/150), combination therapy rose from 22.1% to 27.3%, and the share of records without a documented CDI-specific agent increased from 6.2% to 10.0%. Combination therapy was given preferentially to sicker patients: sepsis was present in 68.5% of patients receiving both agents compared with 53.2% of the others, and their mean comorbidity score was higher (3.0 vs. 2.8). Non-CDI antibiotics (for example, ceftriaxone or meropenem) were documented in 253 episodes (85.8%), and their use tracked concomitant infection: 95.4% of patients with pneumonia and 89.9% of those with sepsis received other antibiotics, compared with 78.0% and 80.3% of those without. Probiotics were recorded in 169 episodes (57.3%). None of the treatment variables differed significantly between periods.

### 2.5. Outcomes and Univariable Predictors of In-Hospital Mortality

Non-survivors were older than survivors (mean 71.9 ± 13.1 vs. 67.9 ± 15.5 years; median 74 vs. 71; *p* = 0.053), and the odds of death rose by 23% per decade of age (OR 1.23, 95% CI 1.02–1.47, *p* = 0.028). Mortality was 26.3% under 65 years, 35.4% at 65 to 79 years and 38.2% at 80 years or older (*p* = 0.246 across groups). Men and women died at similar rates (35.4%, 51/144, vs. 31.8%, 48/151; *p* = 0.509).

**Figure 3:**
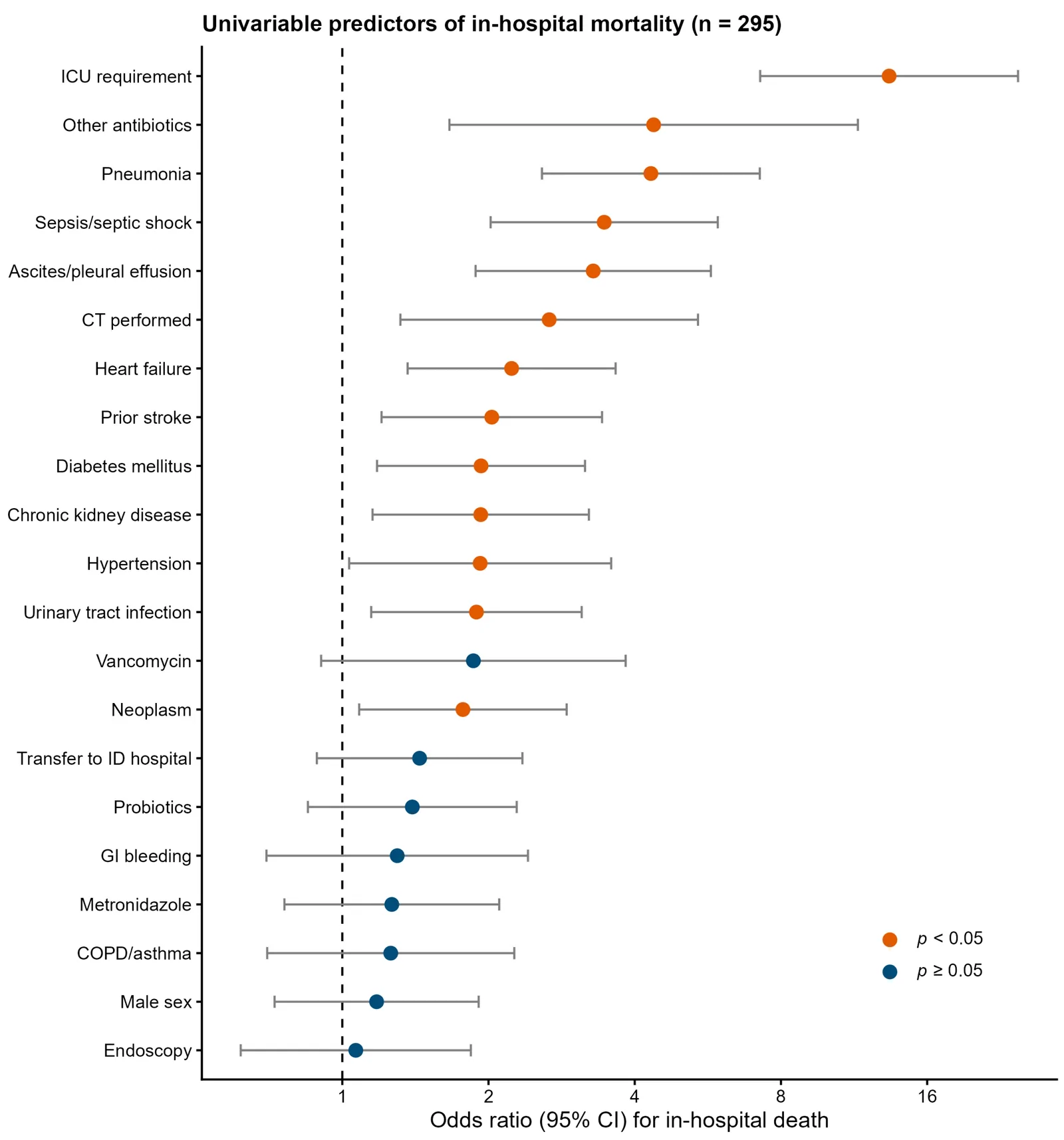
Univariable odds ratios (95% confidence intervals) for in-hospital death, logarithmic scale. Orange markers denote *p* < 0.05 and blue markers *p* ≥ 0.05; horizontal bars show the confidence intervals, and the dashed vertical line marks an odds ratio of 1 (no association).

**Figure 4:**
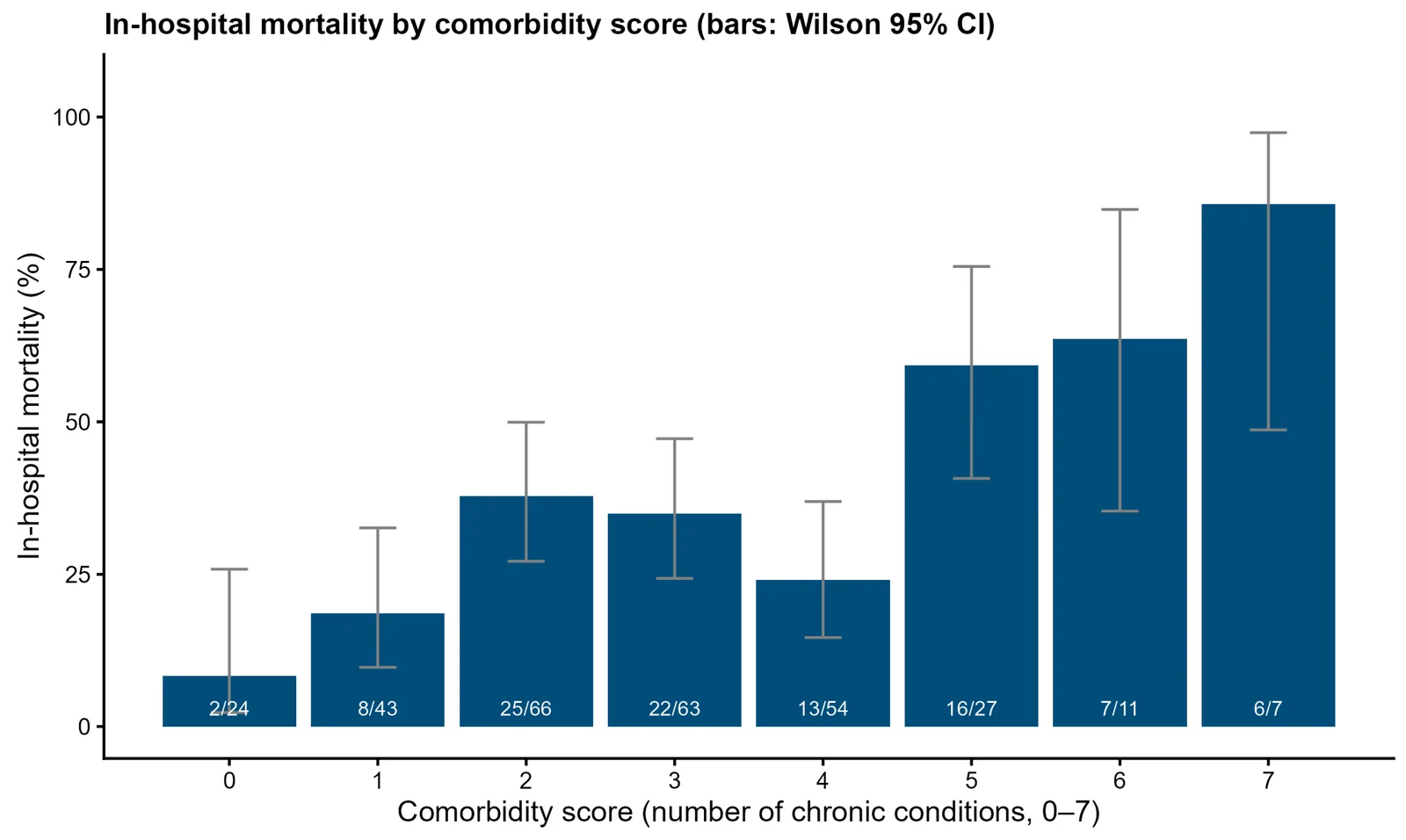
In-hospital mortality by comorbidity score (number of chronic conditions among hypertension, diabetes, chronic kidney disease, prior stroke, neoplasm, heart failure and COPD/asthma). Labels give deaths/patients; error bars are Wilson 95% confidence intervals.

Non-survivors had a higher comorbidity score (3.4 ± 1.7 vs. 2.5 ± 1.5, *p* < 0.001), and each additional chronic condition raised the odds of death by 42% (OR 1.42, 95% CI 1.22–1.66). Every chronic condition except COPD/asthma was associated with death in univariable analysis (Table 3, Figure 3): heart failure (OR 2.23), prior stroke (2.03), diabetes (1.93), chronic kidney disease (1.93), hypertension (1.92) and neoplasm (1.77). Mortality rose from 8.3% (2/24) in patients without comorbidity to 59.3% (16/27), 63.6% (7/11) and 85.7% (6/7) at scores of 5, 6 and 7 (Figure 4). The age-adjusted partial CCI was also higher in non-survivors (6.2 ± 2.6 vs. 4.8 ± 2.5, *p* < 0.001; OR 1.25 per point, 95% CI 1.12–1.38), and mortality rose from 21.1% (15/71) at 0 to 3 points to 54.0% (34/63) at 8 points or more (*p* < 0.001).

**Figure 5:**
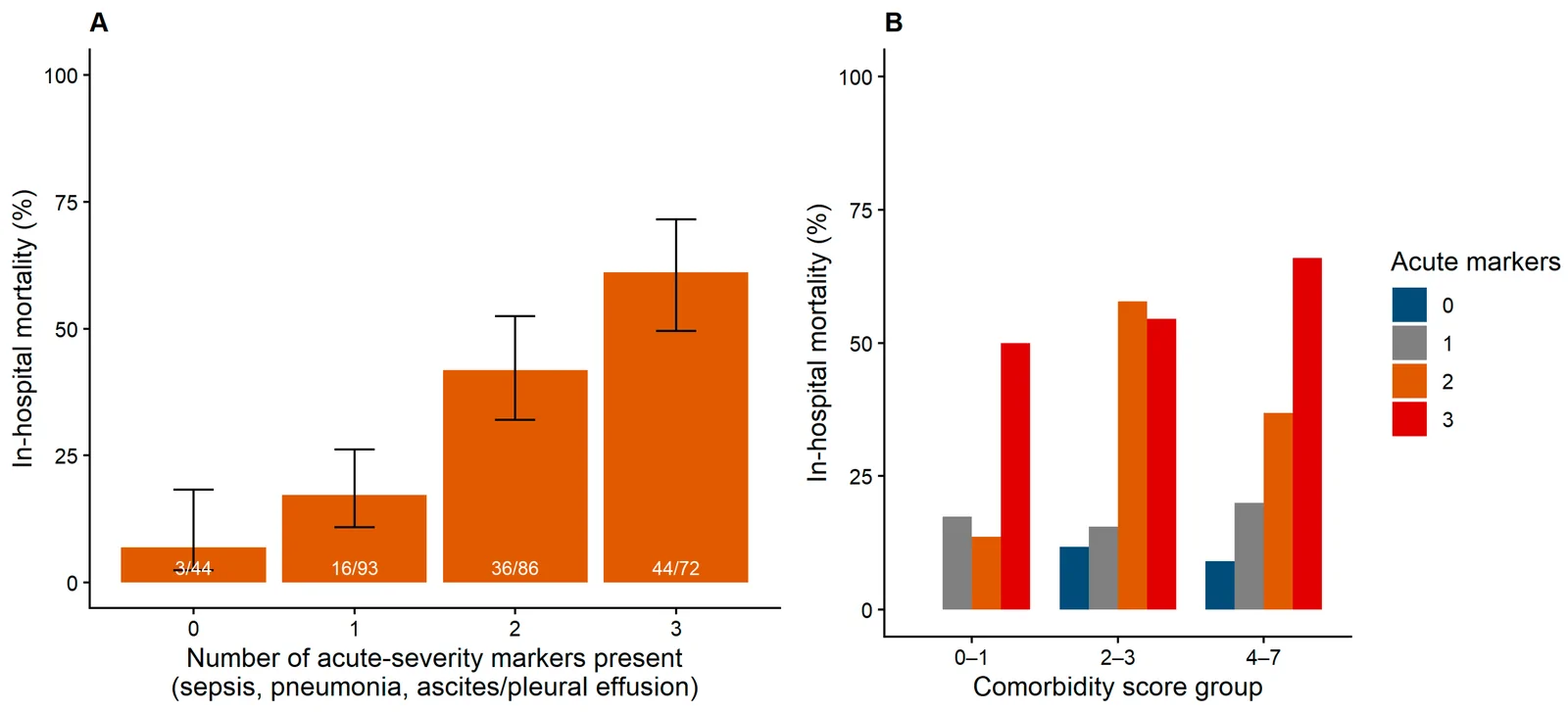
In-hospital mortality by the number of acute-severity markers (sepsis, pneumonia, ascites/pleural effusion). (**A**) Whole cohort, with Wilson 95% confidence intervals; labels give deaths/patients. (**B**) Stratified by comorbidity score group (0–1, 2–3, 4–7 chronic conditions); bar colours give the number of acute-severity markers (0 to 3).

The acute conditions were stronger correlates. Mortality was 51.1% with pneumonia versus 19.5% without (OR 4.32, 95% CI 2.58–7.24), 44.6% with sepsis versus 18.9% without (OR 3.46, 2.02–5.93) and 42.9% with ascites/pleural effusion versus 18.6% without (OR 3.29, 1.88–5.74). Urinary tract infection was associated with a smaller excess (OR 1.89), and gastrointestinal bleeding was associated with none (OR 1.30, *p* = 0.409). Counting the three strongest markers produced a steep gradient: mortality was 6.8% (3/44) with none, 17.2% (16/93) with one, 41.9% (36/86) with two and 61.1% (44/72) with all three (*p* < 0.001; OR 2.78 per additional marker, 95% CI 2.06–3.75) (Figure 5A). The gradient was present at every level of chronic comorbidity: among patients with at most one chronic condition, mortality was 0% (0/16) with no acute marker and 50.0% (3/6) with all three; among those with four or more chronic conditions, it was 9.1% (1/11) and 65.9% (29/44), respectively (Figure 5B).

Among process variables, intensive care requirement was the strongest univariable correlate of death (OR 13.36, 95% CI 7.25–24.61). Performance of computed tomography (OR 2.67) and documentation of non-CDI antibiotics (OR 4.38, 95% CI 1.66–11.52) were also associated with death, consistent with their role as markers of clinical deterioration and of concomitant infection. Neither CDI-specific regimen was associated with mortality after accounting for its indication: crude mortality was 32.2% with vancomycin alone, 43.8% with the combination, 16.7% with metronidazole alone and 29.2% without a documented agent (Table 2). Transfer to the infectious diseases hospital was documented more often among non-survivors (57.6% vs. 48.5%), but the difference was not significant (OR 1.44, 0.89–2.35).

### 2.6. Multivariable Predictors of In-Hospital Mortality

The full model included age, sex, the seven chronic comorbidities and the five acute conditions (14 predictors, 295 patients, 99 deaths, and 7.1 events per variable). All variance inflation factors were below 1.4. Three predictors retained an independent association with death: sepsis (adjusted OR 2.68, 95% CI 1.48–4.86, *p* = 0.001), pneumonia (2.72, 1.47–5.05, *p* = 0.001) and ascites/pleural effusion (2.34, 1.24–4.43, *p* = 0.009). None of the chronic comorbidities remained significant once the acute conditions were included; the adjusted odds ratios for neoplasm (1.55), heart failure (1.46), prior stroke (1.41) and chronic kidney disease (1.21) were attenuated and their confidence intervals crossed unity, and hypertension and diabetes lost their association entirely (Table 4). The full model discriminated moderately (AUC 0.78) with borderline calibration (Hosmer–Lemeshow chi-squared 15.1, 8 df, *p* = 0.058; Nagelkerke-type pseudo-R2 0.18).

**Figure 6:**
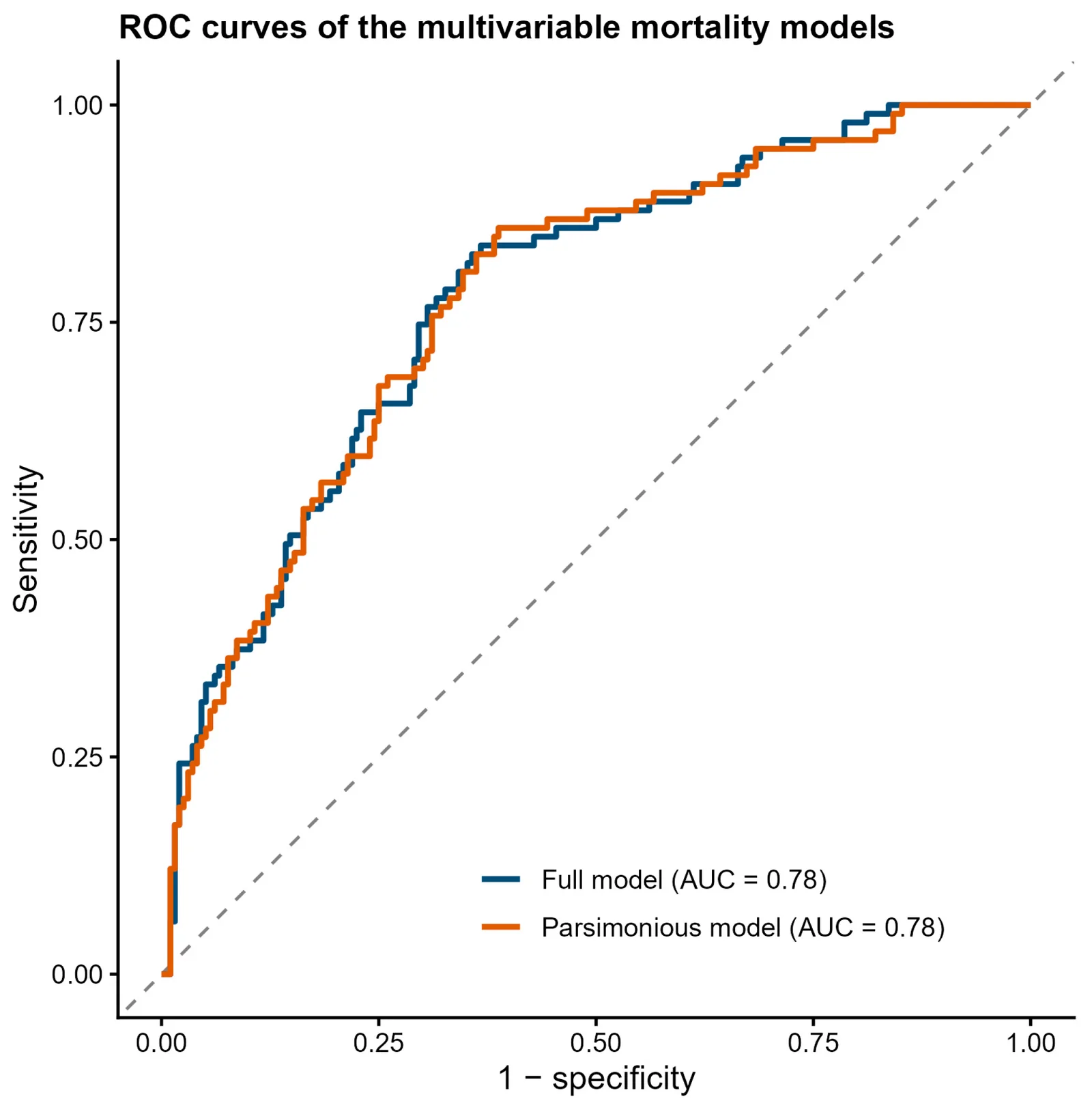
Receiver operating characteristic curves of the full (14-predictor) and parsimonious (LASSO-selected, 9-predictor) logistic regression models for in-hospital death. The dashed diagonal line indicates chance-level discrimination (area under the curve of 0.5).

**Figure 7:**
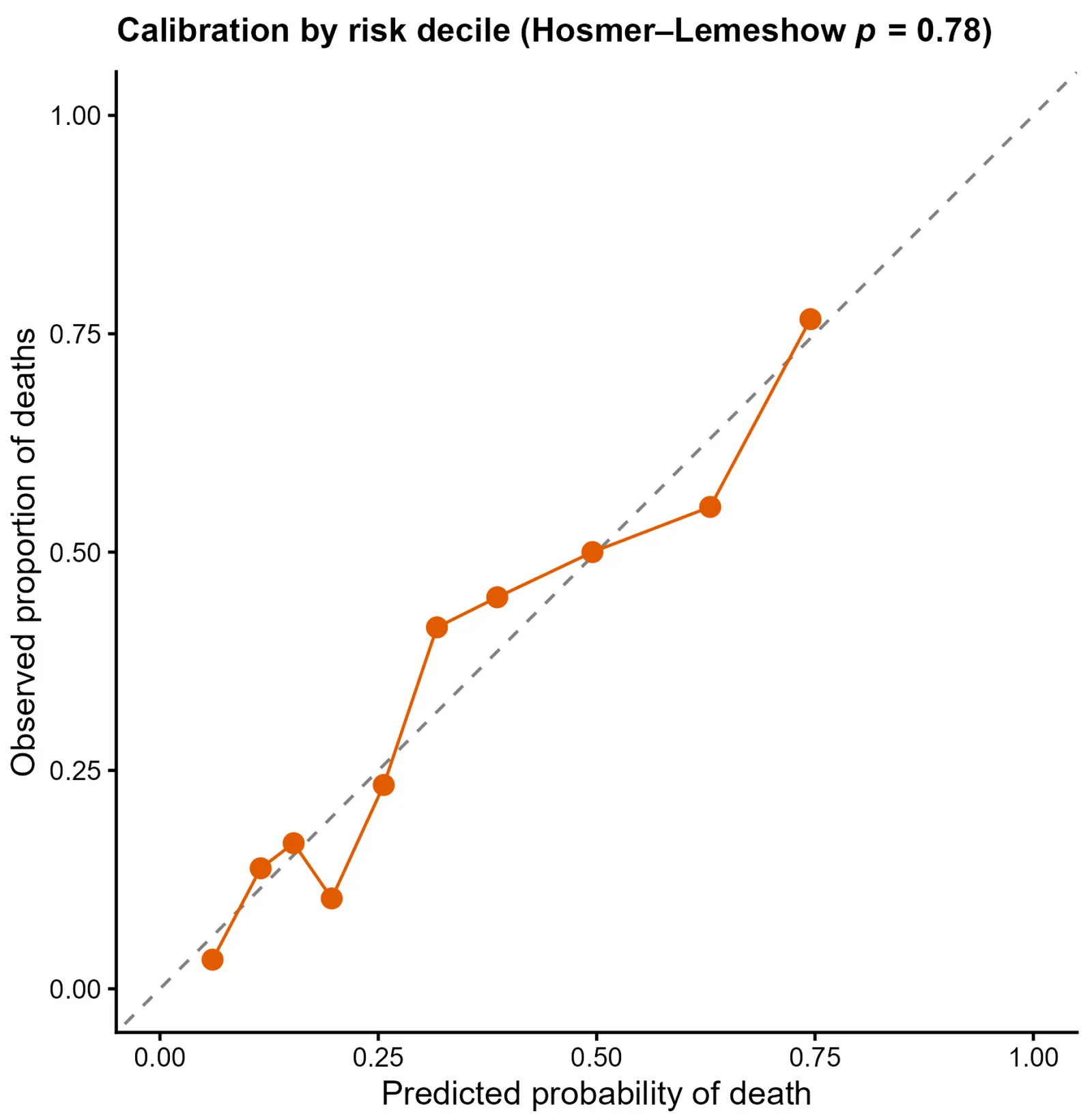
Calibration of the parsimonious model: observed versus predicted probability of in-hospital death across risk deciles (Hosmer–Lemeshow *p* = 0.78). Dots show the observed proportion of deaths against the mean predicted probability in each risk decile and are joined by the solid line; the dashed diagonal line indicates perfect calibration.

LASSO penalised regression with five-fold cross-validation retained nine predictors: age, chronic kidney disease, prior stroke, neoplasm, heart failure, sepsis, pneumonia, urinary tract infection and ascites/pleural effusion. Across 20 repetitions with different random fold assignments, eight of the nine variables were retained every time and urinary tract infection in 15 of 20; hypertension and COPD/asthma entered the model in two and one repetitions respectively, and sex, diabetes and gastrointestinal bleeding were never retained. In the unpenalised refit of the selected variables (11 events per variable), sepsis (adjusted OR 2.68, 95% CI 1.49–4.83, *p* < 0.001), pneumonia (2.53, 1.41–4.56, *p* = 0.002) and ascites/pleural effusion (2.32, 1.24–4.35, *p* = 0.008) remained the only significant predictors (Table 4). The parsimonious model had an apparent AUC of 0.78 (Figure 6); bootstrap resampling (500 replicates) estimated an optimism of 0.03, giving an optimism-corrected AUC of 0.75. Calibration across risk deciles was good (Hosmer–Lemeshow chi-squared 4.8, 8 df, *p* = 0.78; Brier score 0.177) (Figure 7).

Several sensitivity analyses were performed. Adding the study period to the parsimonious model did not change any coefficient (period 2 adjusted OR 1.14, 95% CI 0.65–1.99, *p* = 0.659). Restricting both models to the 292 adult patients gave the same conclusions: in the full model, sepsis (adjusted OR 2.69, 95% CI 1.48–4.88), pneumonia (2.76, 1.49–5.14) and ascites/pleural effusion (2.36, 1.25–4.46) remained the only significant predictors (AUC 0.78), and in the parsimonious model, the corresponding odds ratios were 2.68, 2.54 and 2.32 (AUC 0.78). Adding intensive care requirement as a covariate raised the AUC to 0.85 and was dominated by that variable (adjusted OR 9.30, 95% CI 4.81–17.99); sepsis remained independently associated with death (2.06, 1.08–3.95), whereas the associations of pneumonia (1.73, 0.89–3.35) and ascites/pleural effusion (1.57, 0.78–3.16) were absorbed, as expected for a downstream marker of the same organ dysfunction. Adding documentation of non-CDI antibiotics changed the acute-condition coefficients only marginally (adjusted OR for other antibiotics 2.30, 95% CI 0.80–6.62, *p* = 0.123).

When the seven individual comorbidities were replaced by a single summary of chronic disease, the summary retained a modest independent association that the individual conditions lacked. Adjusted for age, sepsis, pneumonia and ascites/pleural effusion, the odds of death rose by 16% per point of the partial CCI (adjusted OR 1.16, 95% CI 1.02–1.34, *p* = 0.029) and by 21% per point of the unweighted comorbidity score (1.21, 1.01–1.44, *p* = 0.037). The three acute conditions kept adjusted odds ratios between 2.3 and 2.7 in both models, and the comorbidity summary added little to discrimination (AUC 0.776 with the partial CCI vs. 0.763 for age and the three acute conditions alone).

The definition of sepsis was examined in the 144 Period 1 records that had both codings; the manual chart review classified 57 patients as septic and the algorithm 76. In a model with age, sepsis, pneumonia and ascites/pleural effusion, the adjusted odds ratio for sepsis was 2.31 (95% CI 1.04–5.11) with the automated coding and 2.02 (0.93–4.37) with the manual coding, with almost identical discrimination (AUC 0.74 and 0.73); using manual coding for all three acute conditions gave a similar estimate (2.05, 0.94–4.45). The 19 patients flagged only by the algorithm had an intermediate mortality (31.6%, 6/19) between that of manually confirmed sepsis (43.9%, 25/57) and that of patients without sepsis by either definition (20.6%, 14/68).

### 2.7. Toxin A&B Magnitude and Outcome

**Figure 8:**
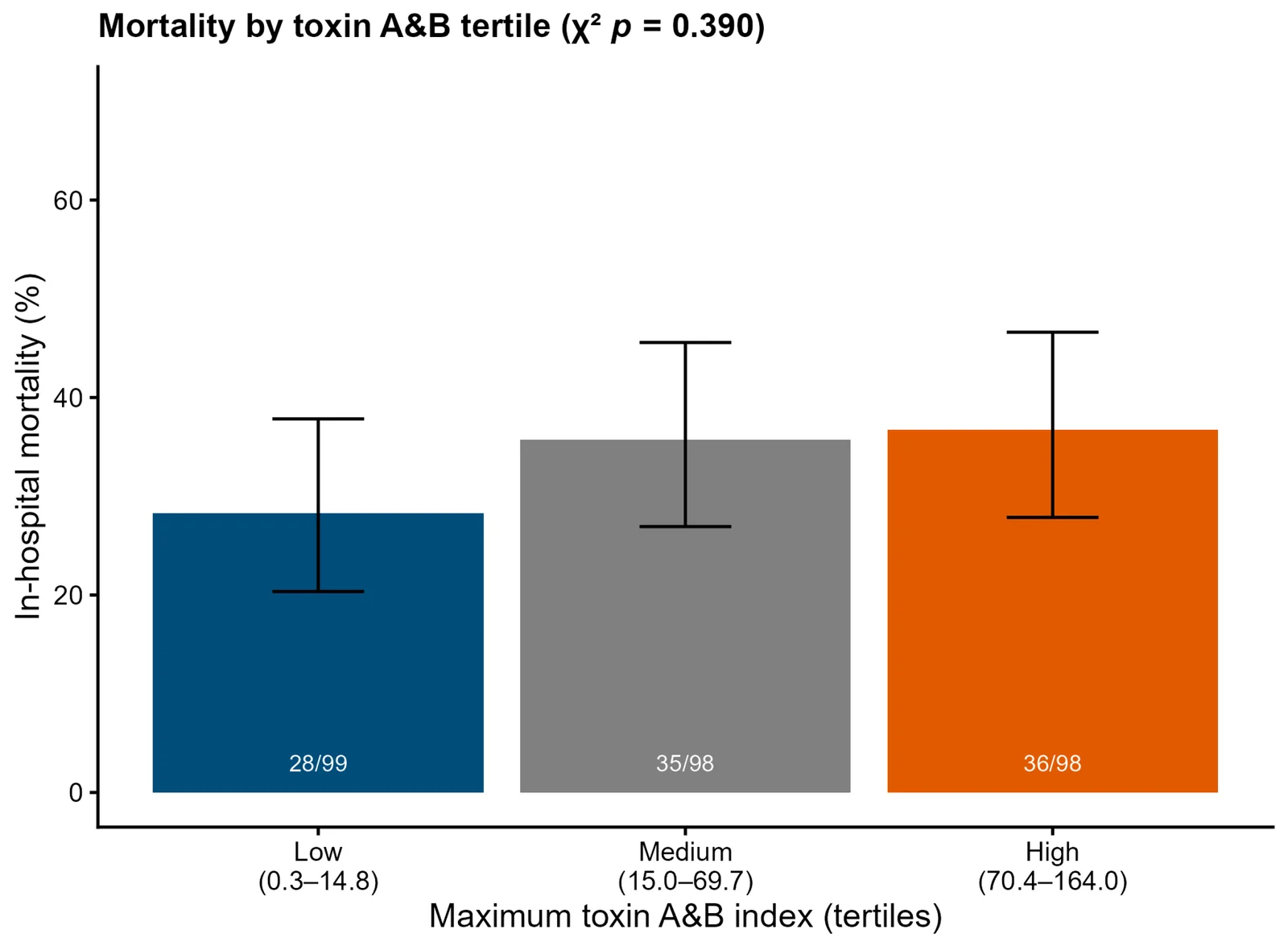
In-hospital mortality by tertile of the maximum toxin A&B index (ranges in parentheses). Bars are coloured by tertile (blue, low; grey, medium; orange, high). Labels give deaths/patients; error bars are Wilson 95% confidence intervals.

The maximum toxin A&B index per patient had a mean of 61.9 ± 61.1 and a median of 35.1 (interquartile range 8.6–113.0). Its distribution was similar in survivors (median 37.9, IQR 7.6–118.8) and non-survivors (median 30.4, IQR 12.5–104.5; *p* = 0.560), and the odds of death did not change with index magnitude (OR 1.00 per 10 units, 95% CI 0.96–1.04). Mortality by tertile was 28.3% (28/99) in the lowest tertile (index 0.3–14.8), 35.7% (35/98) in the middle tertile (15.0–69.7) and 36.7% (36/98) in the highest tertile (70.4–164.0), a small and non-significant gradient (*p* = 0.390; Figure 8). The index was unrelated to age (Spearman rho 0.03, *p* = 0.554) and to the comorbidity score (rho 0.02, *p* = 0.775), and did not differ by intensive care requirement (median 38.9 vs. 32.6, *p* = 0.251) or by the presence of sepsis (*p* = 0.561).

### 2.8. Laboratory Values Available in the Narratives

Numeric laboratory values appeared in the narrative text only when the clinician chose to transcribe them; most records referred to attached laboratory sheets that were not part of the discharge document. Consequently, 68% to 88% of values were missing (Table 5). Among the patients with a recorded value, non-survivors tended to have a lower left ventricular ejection fraction (median 45% vs. 52.5%, *p* = 0.059), higher maximum creatinine (3.4 vs. 2.5 mg/dL, *p* = 0.133) and lower minimum haemoglobin (8.6 vs. 9.9 g/dL, *p* = 0.204). These comparisons are reported for completeness only; the values were not used in the mortality models and cannot be regarded as representative of the cohort.

### 2.9. Agreement Between Automated and Manual Extraction

The agreement between the rule-based algorithm and the pre-existing manual coding of the first period is summarised in Table 6 for 22 binary variables (*n* = 144 records with both codings). Fifteen variables reached the pre-specified ‘high’ grade (accuracy ≥ 90%), two were ‘moderate’ (80 to 89%) and five were ‘low’ (<80%); the median Cohen’s kappa was 0.89. The agreement was perfect for vancomycin and in-hospital death (kappa 1.00) and near-perfect for pneumonia (0.99), metronidazole (0.99), endoscopy (0.98), ascites/pleural effusion (0.96), COPD/asthma (0.95), prior stroke (0.95), computed tomography (0.93), heart failure (0.93), gastrointestinal bleeding (0.89), diabetes (0.89) and hypertension (0.88). The algorithm was highly sensitive for sepsis (100%) but generated 19 false positives (specificity 78.2%, kappa 0.74), the expected behaviour of a keyword rule when a term also appears in differential diagnoses or negated statements. The intensive care requirement was detected with perfect specificity but 81.1% sensitivity (kappa 0.81).

The five low-agreement variables failed for different reasons. For chronic kidney disease the manual reference recorded only two cases, whereas the algorithm flagged 32. A targeted audit of the 31 discordant records showed explicit documentation of chronic kidney disease, KDIGO stage or haemodialysis in the narrative for most of them, so the discordance reflected under-coding in the manual reference; the rule-based value was retained. For neoplasm and urinary tract infection the direction was reversed: the manual reference coded 90 and 113 cases, and the algorithm 52 and 48, giving sensitivities of 57.8% and 39.8%. The most likely explanation is that these diagnoses are expressed through an open vocabulary (site-specific tumour names or histological terms; urinalysis findings) that a fixed keyword list only partly covers. Probiotics (29 false positives, kappa 0.59) and transfer to the infectious diseases hospital (32 false positives, kappa 0.52) were over-called, a pattern consistent with the keyword appearing in the record for reasons other than the fact of interest, such as a recommendation, a prior prescription or a previous admission. Numeric laboratory values were captured less often by the algorithm than by manual abstraction for white blood cell count (17 vs. 67 records) but more often for C-reactive protein (30 vs. 20), procalcitonin (21 vs. 6) and creatinine (38 vs. 29); length of stay could not be extracted at all (0 vs. 11).

## 3. Discussion

In this cohort of 295 CDI episodes treated over 28 months in a Romanian tertiary emergency hospital, one in three patients died before discharge. Patients were old and heavily comorbid, and most presented with, or developed, at least one serious concurrent condition: sepsis, pneumonia or ascites/pleural effusion. These three acute conditions, and not the individual chronic diseases that predispose to them, were the independent predictors of death, and their number stratified mortality from 6.8% in patients with none of them to 61.1% in those with all three; the aggregate burden of chronic disease retained only a modest independent association. The magnitude of the toxin A&B index carried no prognostic information. Over the two periods the population became more comorbid without a measurable change in acute severity, treatment or outcome. Finally, a transparent rule-based extraction pipeline reproduced manual coding with high fidelity for most surveillance variables, and its failure modes were identifiable and specific.

An in-hospital mortality of 33.6% is high compared with recent reports. In a two-centre Swiss cohort of 826 hospitalised patients (2014–2018), mortality at eight weeks was 15.3% [61]; in a Spanish academic hospital (2021–2022), mortality at 12 weeks after treatment was 13.1% in patients younger than 80 years and 35.3% in those aged 80 years or older [62]; and in a nationwide Spanish analysis of 34,557 CDI admissions (2020–2022), in-hospital mortality increased over the study period and was associated with older age, a Charlson index of two or more, intensive care admission, heart failure and malignancy [63]. The Romanian series report lower figures than ours: 9.4% (50/534) in the regional infectious diseases hospital in Iasi [52] and 14.9% among the CDI cases reported in 2024 by 32 hospitals in south-eastern Romania [53]. The closest benchmark is CDI complicated by sepsis, for which a recent multicentre analysis reported a 28-day mortality of 28.8%, similar to that of sepsis of other origin [64]. Earlier European data, such as the 2008 hospital-based survey in which about one in five patients had died three months after diagnosis [9] and the Dutch multicentre cohort that documented a considerable excess of CDI-attributable death [36], point in the same direction. Mortality reported in hospital cohorts varies widely with case mix and follow-up window [10,11,33], and the figure observed here lies at the upper end of the published range, closer to the outcomes reported for older patients and for hospital-acquired disease [19,22]. Several features of the setting explain the difference. The hospital is the regional emergency centre and receives the sickest patients from six counties; two-thirds of the cohort had at least two chronic conditions, 57% had sepsis and 45% required intensive care. Other Romanian hospital series have likewise described a severe case mix in which CDI is the leading reported healthcare-associated infection [52,53,65], and the regional infectious diseases hospital in the same city reported a comparable clinical profile during the pandemic [66]. The mortality reported here is therefore better read as a property of a tertiary, comorbid, sepsis-prone population than of CDI in Romania as a whole. It should also be noted that the 33.6% figure is in-hospital mortality during the index admission only. Patients transferred to the infectious diseases hospital were not followed, so the figure does not reflect 30-day or 90-day mortality, which would be higher still.

The Romanian background helps to interpret these findings. Ribotype 027 emerged in Romanian hospitals in the early 2010s and rapidly became dominant [50,51], and a recent prospective study from north-eastern Romania found binary toxin genes and the tcdC deletion typical of presumptive ribotype 027 in 43 of 50 patients (86%) [67]. A single-centre series documented a rising incidence over the following years; in one hospital, CDI accounted for about one fifth of acute gastroenteritis admissions, with an incidence of 2.76 cases per 10,000 patient-days, and the upward trend was reversed only after isolation measures and restriction of antibiotic prescribing [65]. Recent hospital-based studies from north-eastern and south-eastern Romania describe CDI as the dominant healthcare-associated infection reported to national surveillance, to the point that its visibility may distort the apparent distribution of other infections [52,53]. In western Romania, the regional infectious diseases hospital in Timisoara has reported its experience with CDI during the COVID-19 pandemic, including ribotype data [66], and a tertiary infectious diseases centre has linked the cumulative duration of antibiotic therapy to CDI in hospitalised COVID-19 patients [68]. Community antibiotic consumption in Romania is among the highest in the European Union [54], hospital consumption remains high [69], and *C. difficile* has been isolated from food-producing animals in the country [70]. An ageing population, a large oncological and dialysis caseload and heavy antibiotic pressure are therefore the background against which the present cohort should be read.

The central analytical result is the attenuation of every chronic comorbidity after adjustment for acute conditions. Univariably, heart failure, stroke, diabetes, chronic kidney disease, hypertension and cancer each doubled the odds of death, and the comorbidity score showed a dose–response gradient. In the multivariable model these associations shrank towards unity, whereas sepsis, pneumonia and ascites or pleural effusion each retained an adjusted odds ratio between 2.3 and 2.7. The stratified analysis in Figure 5B makes the mechanism visible: at any level of chronic comorbidity, mortality rose steeply with the number of acute markers, and patients with many chronic conditions but no acute marker fared as well as those with none. Chronic disease therefore appears to act mainly by increasing the likelihood of a severe acute presentation, rather than by adding an independent hazard once that presentation has occurred. The comorbidity summaries qualify this interpretation only slightly: entered as a single term, the partial CCI kept a modest independent association with death (adjusted OR 1.16 per point), which suggests that the accumulated burden of chronic disease adds a small hazard of its own, far smaller than that of any of the three acute conditions.

This pattern agrees with the systematic reviews of mortality risk factors, in which markers of systemic response and organ dysfunction (leucocytosis, hypoalbuminaemia, renal impairment, and haemodynamic instability) outperform demographic and comorbidity variables [32,33], and with the composition of the ATLAS and CARDS scores [34,35]. Sepsis in the present cohort was recorded from the clinicians’ own documentation; although the Sepsis-3 criteria could not be verified retrospectively [71], the label identified a group with more than twice the adjusted odds of death, and the association was of similar size when the stricter manual chart-review coding of the first period was used (adjusted OR 2.02 vs. 2.31 with the automated coding in the same records), although with wider confidence limits. A comparable mortality has been reported for CDI-associated sepsis in a dedicated multicentre analysis [64]. Pneumonia is the most frequent concomitant infection in CDI patients and the one most likely to require systemic antibiotics, which in turn lower the chance of clinical cure and increase recurrence [20,47]; in our data 95% of patients with pneumonia received non-CDI antibiotics. Ascites and pleural effusion are surrogates of hypoalbuminaemia, capillary leak and cardiac or hepatic decompensation, all of which are established severity criteria in current guidance [40,41]. The finding that these three bedside-observable conditions carry most of the prognostic information has a practical corollary: risk stratification at presentation can rely on them where laboratory severity scores cannot be computed, as was the case here.

The individual comorbidities are nonetheless consistent with the literature. Chronic kidney disease increases both incident and recurrent CDI [27]; diabetes is associated with higher incidence and worse hospital outcomes [28,29]; cancer patients with CDI have longer stays and higher mortality [30]; COPD carries an excess burden [26]; and fatty liver disease has recently been added to the list [31]. Their prevalence in this cohort (one-third to three-quarters of patients, depending on the condition) is high in absolute terms and rose further between the two periods. Whether this rise reflects a true change in the CDI population, a change in referral or admission patterns, or better documentation cannot be determined from the present data. The concurrent increase in documented gastrointestinal bleeding and endoscopy suggests that at least part of the change is attributable to case mix, with more patients admitted for other reasons and developing CDI during their stay. To improve comparability with studies that report the Charlson Comorbidity Index [72,73], we also computed a partial CCI from the six Charlson conditions captured by the extraction. Its mean age-adjusted value of 5.3 cannot be compared directly with full Charlson scores, because several Charlson conditions (among them myocardial infarction, peripheral vascular disease, dementia, connective tissue disease, peptic ulcer disease and liver disease) were not extracted, and because diabetes with end-organ damage and metastatic tumours could not be distinguished; the partial index therefore underestimates the full score. Its association with mortality is nonetheless consistent with the Spanish nationwide data, in which a Charlson index of two or more was associated with in-hospital death [63].

The toxin A&B index was unrelated to mortality, to intensive care requirements, to sepsis and to age. This is in line with the mixed literature on stool toxin quantification. An ultrasensitive assay has shown that stool toxin concentrations overlap considerably between symptomatic infection and asymptomatic carriage [38], whereas toxin B PCR cycle threshold, a proxy for bacterial load, predicted poor outcome in a derivation and validation cohort [39]. Detectability of toxin was linked to severity in the ribotype 027 era [19], but detectability and magnitude are different quantities. The index reported by a qualitative or semi-quantitative enzyme immunoassay depends on stool consistency, sampling time relative to symptom onset and the analytical characteristics of the kit, and current diagnostic guidance treats it as a binary result within a two-step algorithm [74]. The present data support that position: the index should not be used to grade severity or to triage patients.

Treatment was dominated by oral vancomycin, which is consistent with the recommendations in force during the study period [40,41,42,43]. Three observations deserve comment. First, metronidazole was still documented in one-third of episodes, alone in 8% and combined with vancomycin in a quarter. Guidelines reserve the combination (intravenous metronidazole with oral vancomycin) for fulminant disease, and the higher prevalence of sepsis among patients who received both agents indicates that this was largely the case; metronidazole monotherapy, which is inferior to vancomycin in randomised trials [45,46], declined from 12% to 4% between periods. Second, fidaxomicin, the preferred first-line agent in the ESCMID 2021 guidance and the IDSA/SHEA 2021 focused update [40,42] because of its lower recurrence rate [44], was not among the extracted variables. Guideline concordance in this cohort can therefore be judged only against the alternative recommendation of oral vancomycin, which was documented in 84% of episodes, and the lack of fidaxomicin use is a gap in guideline-concordant care that the present data cannot quantify further. Third, non-CDI antibiotics were documented in 86% of episodes. Part of this exposure was unavoidable in patients with pneumonia or sepsis, but concomitant antibiotics are the strongest modifiable determinant of treatment failure and recurrence [20,47], and their prevalence in this cohort identifies stewardship during the CDI episode, not only before it, as a target. Probiotics were prescribed in more than half of episodes despite low-certainty evidence [48]. These findings echo the incomplete adherence to guidance observed even in referral centres [49] and the national picture of high antibiotic consumption [54,69].

The second objective was methodological. Romanian hospital records are often stored as narrative discharge documents rather than as structured fields, and few information-extraction tools have been evaluated on Romanian-language clinical narratives, none of them, to our knowledge, for CDI. Rule-based lexical extraction reproduced manual coding with an accuracy of 90% or more for 15 of 22 binary variables and with perfect agreement for the two variables that matter most for surveillance, CDI-specific treatment and death. This level of performance is comparable to the case-detection accuracy reported for rule-based systems in the systematic review by Ford and colleagues [57] and to the comorbidity-extraction results summarised by Wang and colleagues [58], and it was achieved on Romanian-language narratives with a small, inspectable set of keyword rules. The failures were instructive. Variables with a stable, formulaic vocabulary (drug names, death, imaging, endoscopy, and the standard abbreviations for hypertension, diabetes and stroke) were captured almost perfectly. Variables expressed through open vocabularies (tumour names for neoplasm, urinalysis findings for urinary tract infection) were under-called, and variables whose keywords can appear in the record for reasons other than the fact of interest (transfer, probiotics, sepsis) were over-called. In one instance, chronic kidney disease, the discordance exposed an error in the manual reference rather than in the algorithm, illustrating the value of running two independent codings against each other. Numeric laboratory values were the least tractable element, not because of the algorithm but because the values were absent from the narrative.

These results suggest a practical division of labour. Automated extraction can carry the variables with high agreement, and manual verification can be concentrated on the small set of low-agreement variables, which reduces the abstraction burden without sacrificing accuracy. Such hybrid workflows are the norm in electronically assisted surveillance of healthcare-associated infections [56], and they are more accurate than administrative diagnosis codes, which capture CDI incompletely [55]. The next step is to replace or supplement keyword rules with contextual models. Transformer-based and large language models have recently been shown to extract features from free-text records at least as well as rule-based baselines with far less engineering effort [60], and pipelines for microbiology reports have been published [59]. The manually verified first-period dataset produced here provides a labelled corpus against which such models can be evaluated for Romanian clinical text. The full analysis code is provided as Supplementary Material so that the results can be reproduced from the deposited dataset.

The strengths of the study are the consecutive inclusion of laboratory-confirmed episodes over more than two years, the linkage of laboratory and clinical sources, the identical processing of both periods, the explicit quantification of extraction error, the pre-specified exclusion of downstream variables from the primary model, and the internal validation of the prognostic model by bootstrap resampling and calibration testing [75,76]. The limitations are also substantial. The study is single-centre and retrospective, and the population is selected for severe disease by the hospital’s referral role. Diagnosis relied on a toxin A&B enzyme immunoassay alone, without glutamate dehydrogenase screening or nucleic acid amplification testing. Toxin immunoassays are less sensitive than PCR-based methods, so episodes with low toxin concentrations may have been missed and the cohort probably over-represents the toxin-rich, clinically more significant end of the spectrum [74,77]; the absence of PCR and ribotyping also prevented any assessment of hypervirulent lineages such as ribotype 027 or of binary toxin carriage. The two periods differ in length (12 and 16 months) and patient-day denominators were not available, so the episode counts are not incidence rates and do not allow the true dynamics of incidence between periods to be assessed. The binary variables for the second period were extracted automatically and were not verified record by record; the validation results imply that neoplasm and urinary tract infection are under-ascertained and that transfer and probiotic use are over-ascertained in both periods, and the prevalence of neoplasm in particular may be closer to the 62.5% found by manual coding in the first period than to the 39.3% reported here. Sepsis was taken from clinical documentation rather than from Sepsis-3 criteria [71], and a sensitivity analysis based on SOFA scores was not possible because its components were not transcribed systematically; this may have led to both over- and under-diagnosis, although the manual-coding sensitivity analysis gave a similar estimate. Numeric laboratory values were missing for two-thirds or more of patients and length of stay for all, so the ATLAS and CARDS scores could not be computed and albumin, leucocyte count and creatinine could not enter the model [34,35]. The comorbidity score is an unweighted count and is not a validated index, and the partial CCI covers only six Charlson conditions, which limits comparison with studies that report the full index. No recurrence data, prior antibiotic history, proton pump inhibitor use or post-transfer follow-up were available in the source records. The contribution of these modifiable risk factors could therefore not be assessed, and death refers to the index admission only: the 33.6% mortality is in-hospital mortality and does not reflect 30-day, 90-day or longer-term outcomes. The transfer variable records any documentation of transfer to the infectious diseases hospital, including planned transfers, and cannot be used to reconstruct the place of death. The intensive care requirement was treated as a severity marker rather than a predictor; the sensitivity analysis shows that it absorbs part of the effect of pneumonia and effusion, as expected. Three paediatric patients were included because the laboratory series covers the whole hospital; they are shown separately in Table 1, and excluding them did not change the results of either model. With 99 events, the full model had seven events per variable, below the conventional threshold of ten [78]; the parsimonious model met it, but the modest optimism-corrected AUC of 0.75 and the absence of external validation mean that the model is descriptive rather than a clinical prediction tool. Finally, the between-period comparisons were not prespecified and involve 30 tests; the *p*-values in the tables are unadjusted, only some differences survived Holm or Benjamini–Hochberg correction, and all between-period findings should be regarded as exploratory.

For clinicians, the findings support early identification of CDI patients with sepsis, pneumonia or serous effusion as a high-risk group warranting closer monitoring, prompt escalation to guideline-concordant therapy and early involvement of intensive care and infectious diseases specialists, irrespective of the toxin index. For stewardship programmes, the high prevalence of concomitant antibiotics during CDI treatment and the residual use of metronidazole monotherapy identify concrete audit targets, and the rising comorbidity of the CDI population argues for targeting prevention in patients with diabetes, chronic kidney disease and cancer. For surveillance, the extraction pipeline offers a low-cost route to structured CDI data in hospitals without a fully electronic record, and its labelled validation set offers a benchmark for language models. Multicentre extension with patient-day denominators, ribotyping, data on recurrence and on antibiotic and proton pump inhibitor exposure before the episode, 30-day and 90-day follow-up across transfers, and prospective capture of laboratory severity markers would allow the model to be validated externally and converted into a usable score.

## 4. Materials and Methods

### 4.1. Study Design and Setting

We conducted a retrospective observational cohort study with two consecutive inclusion periods at the Pius Brinzeu County Emergency Clinical Hospital in Timisoara, the largest tertiary emergency hospital in western Romania. The hospital serves Timis County and receives referrals from the neighbouring counties of the region, as reflected in the residence distribution of the cohort. Patients with CDI are managed on the admitting ward or in intensive care and are transferred, when clinically appropriate and when capacity allows, to the regional Victor Babes Hospital for Infectious Diseases in the same city. The overall study window ran from 1 September 2023 to 31 December 2025. Period 1 ran from 1 September 2023 to 1 September 2024 (12 months) and Period 2 from 2 September 2024 to 31 December 2025 (16 months); both periods refer to the date of the toxin A&B determination that established the laboratory diagnosis of CDI. The two periods correspond to two successive data-collection cycles; the first had been coded by hand before the present study, and the second was coded with the automated pipeline. The study is reported in line with the Strengthening the Reporting of Observational Studies in Epidemiology (STROBE) recommendations for cohort studies and with the applicable items of the Transparent Reporting of a multivariable prediction model for Individual Prognosis Or Diagnosis (TRIPOD) statement for the multivariable model [75,79].

### 4.2. Case Definition and Eligibility

The starting point was the laboratory register of all *C. difficile* toxin A&B determinations performed in the hospital during the study window. Toxins A and B were detected in unformed (diarrhoeal) stool specimens with an chemiluminescence immunoassay (LIAISON^®^ *C. difficile* Toxins A&B; DiaSorin, Stillwater, MN, USA), which reports a numerical index; a determination was classified as positive at an index of 1.0 or higher, the threshold used by the laboratory. A CDI episode was defined as a patient with at least one positive determination and a clinical record documenting diarrhoeal illness treated as CDI, in keeping with the ESCMID definition of CDI as a compatible clinical picture with microbiological evidence of toxin-producing *C. difficile* [40,74]. Two additional episodes in which the maximum index was below the threshold but CDI was explicitly diagnosed and treated in the record were retained. Patients whose clinical record could not be retrieved were not included. Each patient contributed at most one episode per period; a patient with positive determinations in both periods therefore contributed one episode to each period and was counted twice (*n* = 23). Within a period, the maximum toxin index across all determinations for that patient was used as the toxin variable, and the number of determinations was recorded. No age restriction was applied, so three patients under 18 years appear in the cohort; they are shown as a separate age group in Table 1 and were excluded in a sensitivity analysis.

### 4.3. Data Sources and Record Linkage

Five source files were integrated: one spreadsheet containing the manual coding of Period 1; two word-processor files containing the full free-text clinical records (one per period); and two PDF laboratory reports listing every toxin A&B determination with test date, quantitative index, national personal identification number (CNP) and county of residence. Sex and year of birth were derived from the CNP. Each clinical record carried a numeric identifier, and a composite period-identifier code was used as the unique episode key. The laboratory results were linked to the clinical records by patient name using exact matching first, then prefix matching, then fuzzy string matching for residual records. Patient names were removed from the analysis dataset after linkage. The anonymised dataset, the variable dictionary and the validation sheets are provided as Supplementary Table S1.

### 4.4. Variable Extraction

Thirty variables were defined a priori and grouped as demographic (age, sex, county), laboratory (maximum toxin index, number of determinations), chronic comorbidities (hypertension, diabetes mellitus, chronic kidney disease, history of stroke, malignancy of any site, heart failure, chronic obstructive pulmonary disease or asthma), acute conditions (sepsis or septic shock, upper or lower gastrointestinal bleeding, pneumonia or pulmonary infiltrate, urinary tract infection including pyelonephritis and urosepsis, ascites or pleural effusion), investigations (computed tomography, gastroscopy or colonoscopy), treatments (vancomycin, metronidazole, probiotics, other antibiotics), numeric laboratory values where transcribed in the narrative (maximum white blood cell count, C-reactive protein, procalcitonin, creatinine and urea, minimum haemoglobin, left ventricular ejection fraction), and outcomes (intensive care management, transfer to the infectious diseases hospital, in-hospital death). Length of stay was also targeted but could not be extracted. Full definitions and coding are given in the Data_Dictionary sheet of Supplementary Table S1.

Binary variables were extracted from the narrative with a rule-based lexical algorithm. For each variable a list of Romanian keywords, word stems and the abbreviations used in the hospital’s records (for example, arterial hypertension (HTA, Romanian *hipertensiune arterială*), diabetes mellitus (DZ, *diabet zaharat*), chronic kidney disease (BCR, *boală cronică de rinichi*), stroke (AVC, *accident vascular cerebral*), chronic obstructive pulmonary disease (BPOC, *bronhopneumopatie obstructivă cronică*), upper or lower gastrointestinal bleeding (HDS/HDI, *hemoragie digestivă superioară/inferioară*), urinary tract infection (ITU, *infecție de tract urinar*) and the anaesthesia and intensive care unit (ATI, *Anestezie și Terapie Intensivă*)) was compiled, and a variable was coded present when any term of its list occurred in the record. Sepsis was therefore coded from the clinicians’ own documentation: the variable was set to present when the Romanian terms for sepsis or septic shock appeared in the record, and no physiological criteria were applied, because the components of the Sequential Organ Failure Assessment (SOFA) score (PaO_2_/FiO_2_ ratio, platelet count, bilirubin, mean arterial pressure or vasopressor use, Glasgow Coma Scale and creatinine or urine output) were not transcribed systematically in the narratives. Numeric values were captured with pattern matching anchored on the analyte name. The same rules were applied to both periods so that any extraction error is shared between them and does not bias the between-period comparison. Two derived variables were computed: an unweighted comorbidity score equal to the number of the seven chronic conditions present (range 0 to 7), and an acute-severity count equal to the number of the three acute conditions later found to be independent predictors (sepsis, pneumonia, and ascites/pleural effusion; range 0 to 3). The latter was defined after the multivariable analysis and is presented as a descriptive summary, not as a validated score. To allow comparison with studies that use the Charlson Comorbidity Index (CCI) [72,73], a partial CCI was computed from the six Charlson conditions captured by the extraction, with the original weights: heart failure, cerebrovascular disease (prior stroke), chronic pulmonary disease (COPD/asthma) and diabetes mellitus 1 point each, and renal disease (chronic kidney disease) and malignancy (neoplasm) 2 points each (range 0 to 8). Diabetes with end-organ damage and metastatic disease could not be distinguished and was scored at the lower weight; all chronic kidney disease was scored as renal disease, and hypertension, which has no Charlson weight, was not counted. An age-adjusted version added 1 point for each decade from 50 years (50 to 59 years, 1 point; 80 years or older, 4 points), as in the combined age-comorbidity index [73].

### 4.5. Validation of the Automated Extraction

The algorithm was validated against the manual coding of Period 1, which pre-existed the algorithm and was produced from the same records independently of it (144 records with both codings). For each binary variable we computed the numbers of true positives, true negatives, false positives and false negatives, and from these the accuracy, sensitivity, specificity, positive and negative predictive value and Cohen’s kappa, treating the manual coding as the reference. Reliability was graded a priori as high (accuracy ≥ 90%), moderate (80 to 89%) or low (<80%); low-grade variables were flagged for manual verification. Where the algorithm and the reference disagreed systematically, the discordant records were re-read. For chronic kidney disease this audit showed that the narrative contained explicit documentation (chronic kidney disease, KDIGO stage or haemodialysis) in most records flagged only by the algorithm, and the automated value was retained. For all other variables the automated values were used for both periods, to preserve comparability, and the validation table is reported so that readers can judge each variable.

### 4.6. Statistical Analysis

Continuous variables are summarised as mean ± standard deviation together with median and range or interquartile range, and categorical variables as counts and percentages with the full cohort as the denominator. Between-period comparisons used the chi-squared test without continuity correction for categorical variables (Fisher’s exact test when any expected cell count was below five) and the Mann–Whitney U test for continuous variables, because age, the toxin index and the scores were not normally distributed. Associations with in-hospital death were first examined univariably as odds ratios with Wald 95% confidence intervals from 2 × 2 tables, and as odds ratios per unit from single-predictor logistic regression for age, the comorbidity score, the acute-severity count and the toxin index.

The primary multivariable model was a binary logistic regression of in-hospital death on baseline and at-presentation characteristics: age, sex, the seven chronic comorbidities and the five acute conditions. Intensive care requirements, investigations and treatments were excluded from the primary model because they are consequences of, or indications determined by, disease severity. Multicollinearity was assessed with variance inflation factors. A parsimonious model was derived by L1-penalised (LASSO) logistic regression [80] on standardised predictors, with the penalty chosen by five-fold cross-validation over 50 values on a logarithmic grid to minimise the cross-validated log-loss; the stability of the selected set was checked by repeating the procedure with 20 different random fold assignments. The selected variables were then refitted without penalty to obtain adjusted odds ratios. Discrimination was quantified by the area under the receiver operating characteristic curve (AUC), and its optimism was estimated by 500 bootstrap resamples (refitting the model in each resample and evaluating it on the original data) to give an optimism-corrected AUC [76]. Calibration was assessed with the Hosmer–Lemeshow test over risk deciles, a calibration plot and the Brier score. Events per variable were reported against the conventional minimum of ten [78]. Sensitivity analyses added the study period, intensive care requirement or non-CDI antibiotics to the parsimonious model, and repeated the full and parsimonious models in adults only. The partial CCI (with and without age points) and the unweighted score were analysed as continuous variables, univariably and as a single comorbidity term in a model with age and the three independent acute conditions. The sepsis variable was examined in the Period 1 records by refitting a model with age, sepsis, pneumonia and ascites/pleural effusion with the manual chart-review coding of sepsis in place of the automated coding. Mortality by toxin tertile (cut points rounded to the one-decimal reporting precision of the assay) and by number of acute markers was compared with the chi-squared test, and correlations with Spearman’s rho. Exploratory comparisons of laboratory values between survivors and non-survivors used the Mann–Whitney U test on available cases only. All tests were two-sided, with *p* < 0.05 considered significant. The *p*-values in the tables are unadjusted; because the between-period comparisons involved 30 tests and were not prespecified, they were additionally adjusted with the Holm procedure and with the Benjamini–Hochberg false discovery rate [81], and they are interpreted as exploratory. Analyses were performed in R version 4.5.1 (R Foundation for Statistical Computing, Vienna, Austria) with the packages readxl 1.4.5, glmnet 5.0 (penalised regression), pROC 1.19.1 (receiver operating characteristic analysis), ResourceSelection 0.3.6 (Hosmer–Lemeshow test), car 3.1.5 (variance inflation factors) and ggplot2 4.0.3 (figures). The complete R script that reproduces every table and figure from Supplementary Table S1 is provided as Supplementary File S1.

### 4.7. Ethics

The study was approved by the Ethics Committee of the Pius Brinzeu County Emergency Clinical Hospital, Timisoara (protocol 538, approved on 24 November 2025) and was conducted in accordance with the Declaration of Helsinki. Because the design was retrospective and the analysis dataset was anonymised after linkage, the requirement for individual informed consent was waived.

## 5. Conclusions

CDI in this Romanian tertiary emergency hospital affected an old, heavily comorbid population and carried an in-hospital mortality of one in three (33.6%) during the index admission. The individual chronic conditions that predispose to severe disease lost their independent prognostic weight once the acute presentation was taken into account: sepsis, pneumonia and ascites or pleural effusion were the independent predictors of death, and their number graded in-hospital mortality from 6.8% in patients with none of them to 61.1% in those with all three. The magnitude of the toxin A&B index was not associated with in-hospital death (OR 1.00 per 10 index units, 95% CI 0.96–1.04). Over the 28-month study window the population became more comorbid, with more diabetes, chronic kidney disease and hypertension, while treatment remained vancomycin-based with substantial residual metronidazole and near-universal exposure to other antibiotics. A rule-based extraction pipeline reproduced manual coding for most surveillance variables, and its documented weaknesses point to where manual verification or contextual language models are needed. Together, the clinical and methodological findings support early bedside risk stratification of CDI patients and offer a reproducible route to multicentre CDI surveillance in partially digitised health systems.

## Figures and Tables

**Table 1:** Characteristics of the 295 CDI episodes, overall and by study period.

Variable	Total (*n* = 295)	2023–2024 (*n* = 145)	2024–2025 (*n* = 150)	*p*	Test
Age, years	69.2 ± 14.8; 72 [5–95]	68.4 ± 15.7; 72 [5–94]	70.0 ± 13.8; 72 [15–95]	0.698	Mann–Whitney U
Age < 18	3 (1.0%)	2 (1.4%)	1 (0.7%)	0.523	Fisher exact
Age 18–64	77 (26.1%)	35 (24.1%)	42 (28.0%)		
Age 65–79	147 (49.8%)	78 (53.8%)	69 (46.0%)		
Age ≥ 80	68 (23.1%)	30 (20.7%)	38 (25.3%)		
Male sex	144/295 (48.8%)	72/145 (49.7%)	72/150 (48.0%)	0.776	χ^2^
Female sex	151/295 (51.2%)	73/145 (50.3%)	78/150 (52.0%)	0.776	χ^2^
*Chronic comorbidities*					
Comorbidity score (0–7)	2.8 ± 1.7; 3 [0–7]	2.5 ± 1.5; 2 [0–6]	3.1 ± 1.8; 3 [0–7]	0.002	Mann–Whitney U
Partial CCI, age-adjusted (0–11)	5.3 ± 2.6; 5 [0–11]	4.8 ± 2.4; 5 [0–10]	5.7 ± 2.7; 6 [0–11]	0.004	Mann–Whitney U
Hypertension	226/295 (76.6%)	103/145 (71.0%)	123/150 (82.0%)	0.026	χ^2^
Diabetes mellitus	115/295 (39.0%)	44/145 (30.3%)	71/150 (47.3%)	0.003	χ^2^
Chronic kidney disease	91/295 (30.8%)	32/145 (22.1%)	59/150 (39.3%)	0.001	χ^2^
Prior stroke	84/295 (28.5%)	41/145 (28.3%)	43/150 (28.7%)	0.941	χ^2^
Neoplasm	116/295 (39.3%)	52/145 (35.9%)	64/150 (42.7%)	0.232	χ^2^
Heart failure	137/295 (46.4%)	63/145 (43.4%)	74/150 (49.3%)	0.311	χ^2^
COPD/asthma	61/295 (20.7%)	25/145 (17.2%)	36/150 (24.0%)	0.152	χ^2^
*Acute conditions*					
Sepsis/septic shock	168/295 (56.9%)	76/145 (52.4%)	92/150 (61.3%)	0.122	χ^2^
GI bleeding	52/295 (17.6%)	17/145 (11.7%)	35/150 (23.3%)	0.009	χ^2^
Pneumonia	131/295 (44.4%)	68/145 (46.9%)	63/150 (42.0%)	0.397	χ^2^
Urinary tract infection	105/295 (35.6%)	49/145 (33.8%)	56/150 (37.3%)	0.525	χ^2^
Ascites/pleural effusion	182/295 (61.7%)	92/145 (63.4%)	90/150 (60.0%)	0.542	χ^2^
Acute-severity count (0–3)	1.6 ± 1.0; 2 [0–3]	1.6 ± 1.0; 2 [0–3]	1.6 ± 1.1; 2 [0–3]	0.983	Mann–Whitney U
*Investigations*					
CT performed	235/295 (79.7%)	122/145 (84.1%)	113/150 (75.3%)	0.060	χ^2^
Endoscopy	78/295 (26.4%)	26/145 (17.9%)	52/150 (34.7%)	0.001	χ^2^
*Treatment*					
Vancomycin	247/295 (83.7%)	118/145 (81.4%)	129/150 (86.0%)	0.282	χ^2^
Metronidazole	97/295 (32.9%)	50/145 (34.5%)	47/150 (31.3%)	0.565	χ^2^
Probiotics	169/295 (57.3%)	78/145 (53.8%)	91/150 (60.7%)	0.233	χ^2^
Other antibiotics	253/295 (85.8%)	126/145 (86.9%)	127/150 (84.7%)	0.584	χ^2^
*Outcomes*					
ICU requirement	134/295 (45.4%)	60/145 (41.4%)	74/150 (49.3%)	0.170	χ^2^
Transfer to ID hospital	152/295 (51.5%)	70/145 (48.3%)	82/150 (54.7%)	0.272	χ^2^
In-hospital death	99/295 (33.6%)	45/145 (31.0%)	54/150 (36.0%)	0.367	χ^2^
*Toxin A&B testing*					
Toxin A&B index, maximum	61.9 ± 61.1; 35.1 [0.3–164.0]	64.7 ± 59.0; 41.6 [2.9–164.0]	59.1 ± 63.1; 28.7 [0.3–164.0]	0.091	Mann–Whitney U
Toxin determinations per patient	1.47 ± 0.88; 1 [1–6]	1.41 ± 0.83; 1 [1–6]	1.54 ± 0.92; 1 [1–5]	0.157	Mann–Whitney U

Data are n/N (%) or mean ± SD; median [range]. Percentages use the full cohort as the denominator. Comorbidity score: number of the seven chronic conditions present. Acute-severity count: number of sepsis, pneumonia and ascites/pleural effusions present. Toxin A&B index: maximum value per patient (laboratory positivity threshold 1.0). Between-period *p*-values from the chi-squared test without continuity correction (Fisher exact test where indicated) or the Mann–Whitney U test. CCI, Charlson Comorbidity Index; CDI, *Clostridioides difficile* infection; COPD, chronic obstructive pulmonary disease; CT, computed tomography; GI, gastrointestinal; ICU, intensive care unit; ID, infectious diseases; SD, standard deviation. Age groups: the three patients younger than 18 years are shown separately (Fisher’s exact test across the four age groups). Partial CCI: weighted sum of the six Charlson conditions captured (heart failure, prior stroke, COPD/asthma and diabetes mellitus 1 point each; chronic kidney disease and neoplasm 2 points each) plus age points (Section 4.4). *p*-values are unadjusted and the between-period comparisons are exploratory; Holm- and Benjamini–Hochberg-adjusted values are given in Supplementary Table S1 (sheet Multiplicity).

**Table 2:** CDI-specific treatment regimens by study period, with intensive care requirements and in-hospital mortality by regimen.

Regimen Documented	Total (*n* = 295)	2023–2024 (*n* = 145)	2024–2025 (*n* = 150)	ICU Requirement	In-Hospital Death
Vancomycin alone	174 (59.0%)	86 (59.3%)	88 (58.7%)	85/174 (48.9%)	56/174 (32.2%)
Vancomycin + metronidazole	73 (24.7%)	32 (22.1%)	41 (27.3%)	36/73 (49.3%)	32/73 (43.8%)
Metronidazole alone	24 (8.1%)	18 (12.4%)	6 (4.0%)	4/24 (16.7%)	4/24 (16.7%)
Neither documented	24 (8.1%)	9 (6.2%)	15 (10.0%)	9/24 (37.5%)	7/24 (29.2%)

Distribution of regimens between periods: chi-squared *p* = 0.036. Crude mortality by regimen is confounded by indication (combination therapy was used in sicker patients) and was not modelled. ICU, intensive care unit.

**Table 3:** Univariable associations with in-hospital death.

Variable	Deaths/N, Exposed (%)	Deaths/N, Unexposed (%)	OR (95% CI)	*p*
Female sex	48/151 (31.8%)	51/144 (35.4%)	0.85 (0.52–1.38)	0.509
Male sex	51/144 (35.4%)	48/151 (31.8%)	1.18 (0.73–1.91)	0.509
Hypertension	83/226 (36.7%)	16/69 (23.2%)	1.92 (1.03–3.58)	0.037
Diabetes mellitus	49/115 (42.6%)	50/180 (27.8%)	1.93 (1.18–3.16)	0.009
Chronic kidney disease	40/91 (44.0%)	59/204 (28.9%)	1.93 (1.15–3.22)	0.012
Prior stroke	38/84 (45.2%)	61/211 (28.9%)	2.03 (1.20–3.43)	0.007
Neoplasm	48/116 (41.4%)	51/179 (28.5%)	1.77 (1.08–2.90)	0.022
Heart failure	59/137 (43.1%)	40/158 (25.3%)	2.23 (1.36–3.65)	0.001
COPD/asthma	23/61 (37.7%)	76/234 (32.5%)	1.26 (0.70–2.26)	0.441
Sepsis/septic shock	75/168 (44.6%)	24/127 (18.9%)	3.46 (2.02–5.93)	<0.001
GI bleeding	20/52 (38.5%)	79/243 (32.5%)	1.30 (0.70–2.41)	0.409
Pneumonia	67/131 (51.1%)	32/164 (19.5%)	4.32 (2.58–7.24)	<0.001
Urinary tract infection	45/105 (42.9%)	54/190 (28.4%)	1.89 (1.15–3.11)	0.012
Ascites/pleural effusion	78/182 (42.9%)	21/113 (18.6%)	3.29 (1.88–5.74)	<0.001
CT performed	88/235 (37.4%)	11/60 (18.3%)	2.67 (1.32–5.40)	0.005
Endoscopy	27/78 (34.6%)	72/217 (33.2%)	1.07 (0.62–1.84)	0.818
Vancomycin	88/247 (35.6%)	11/48 (22.9%)	1.86 (0.90–3.83)	0.088
Metronidazole	36/97 (37.1%)	63/198 (31.8%)	1.26 (0.76–2.10)	0.366
Probiotics	62/169 (36.7%)	37/126 (29.4%)	1.39 (0.85–2.29)	0.188
Other antibiotics	94/253 (37.2%)	5/42 (11.9%)	4.37 (1.66–11.52)	0.001
ICU requirement	82/134 (61.2%)	17/161 (10.6%)	13.36 (7.25–24.61)	<0.001
Transfer to ID hospital	57/152 (37.5%)	42/143 (29.4%)	1.44 (0.89–2.35)	0.139
Age (per 10 years)			1.23 (1.02–1.47)	0.028
Comorbidity score (per point)			1.42 (1.22–1.66)	<0.001
Acute-severity count (per condition)			2.78 (2.06–3.75)	<0.001
Toxin A&B index (per 10 units)			1.00 (0.96–1.04)	0.892

OR, odds ratio with a Wald 95% confidence interval from 2 × 2 tables (chi-squared *p*-value), or per-unit odds ratio from single-predictor logistic regression for the continuous variables (Wald *p*-value). COPD, chronic obstructive pulmonary disease; CT, computed tomography; GI, gastrointestinal; ICU, intensive care unit; ID, infectious diseases. Female and male sex are shown as complementary comparisons, each against the other sex.

**Table 4:** Multivariable logistic regression for in-hospital death: full model and LASSO-selected parsimonious model.

Predictor	Full Model aOR (95% CI)	*p*	Parsimonious Model aOR (95% CI)	*p*
Age (per year)	1.01 (0.99–1.03)	0.299	1.01 (0.99–1.03)	0.243
Female sex	1.00 (reference)		not selected	
Male sex (vs female)	0.94 (0.53–1.68)	0.840	not selected	
Hypertension	1.07 (0.50–2.29)	0.871	not selected	
Diabetes mellitus	0.96 (0.53–1.76)	0.898	not selected	
Chronic kidney disease	1.21 (0.63–2.30)	0.564	1.21 (0.64–2.27)	0.562
Prior stroke	1.41 (0.75–2.66)	0.289	1.44 (0.78–2.67)	0.241
Neoplasm	1.55 (0.88–2.76)	0.131	1.50 (0.85–2.64)	0.162
Heart failure	1.46 (0.80–2.67)	0.221	1.44 (0.79–2.61)	0.230
COPD/asthma	0.77 (0.38–1.55)	0.470	not selected	
Sepsis/septic shock	2.68 (1.48–4.86)	0.001	2.68 (1.49–4.83)	<0.001
GI bleeding	0.90 (0.44–1.88)	0.786	not selected	
Pneumonia	2.72 (1.47–5.05)	0.001	2.53 (1.41–4.56)	0.002
Urinary tract infection	1.13 (0.62–2.05)	0.687	1.12 (0.62–2.01)	0.712
Ascites/pleural effusion	2.34 (1.24–4.43)	0.009	2.32 (1.24–4.35)	0.008
Events/predictors (EPV)	99/14 (7.1)		99/9 (11.0)	
AUC (apparent)	0.78		0.78	
AUC (optimism-corrected, 500 bootstraps)			0.75	
Hosmer–Lemeshow chi-squared (8 df), *p*	15.1, 0.058		4.8, 0.777	
Brier score			0.177	
Maximum VIF	1.28		1.22	

aOR, adjusted odds ratio; AUC, area under the receiver operating characteristic curve; CI, confidence interval; COPD, chronic obstructive pulmonary disease; EPV, events per variable; GI, gastrointestinal; LASSO, least absolute shrinkage and selection operator (L1 penalty chosen by 5-fold cross-validation; eight of the nine variables were selected in all 20 repetitions with different fold assignments and urinary tract infection in 15 of 20); VIF, variance inflation factor. Intensive care requirements, investigations and treatments were excluded from both models as downstream or indication-confounded variables.

**Table 5:** Laboratory values transcribed in the clinical narratives, by survival status (available cases only).

Parameter	*n* Available	Missing (%)	All Patients, Median [IQR]	Non-Survivors, Median [IQR]	Survivors, Median [IQR]	*p*
WBC, max (/uL)	39	87	10,980.0 [7220.0–16,585.0]	10,790.0 [3862.5–14,890.0] (*n* = 22)	11,270.0 [7600.0–17,500.0] (*n* = 17)	0.470
CRP, max (mg/L)	75	75	70.9 [33.0–187.5]	63.0 [30.7–200.9] (*n* = 35)	76.0 [39.5–186.2] (*n* = 40)	0.937
Procalcitonin (ng/mL)	36	88	4.0 [2.0–5.0]	4.0 [2.0–5.0] (*n* = 17)	2.1 [2.0–5.0] (*n* = 19)	0.519
Creatinine, max (mg/dL)	94	68	3.0 [1.5–5.4]	3.4 [2.0–6.6] (*n* = 36)	2.5 [1.3–4.4] (*n* = 58)	0.133
Urea, max (mg/dL)	79	73	176.0 [103.5–253.5]	179.0 [116.0–257.0] (*n* = 35)	144.0 [104.8–247.5] (*n* = 44)	0.456
Haemoglobin, min (g/dL)	77	74	9.2 [7.5–10.6]	8.6 [7.5–10.2] (*n* = 36)	9.9 [8.2–11.2] (*n* = 41)	0.204
LVEF (%)	53	82	50.0 [45.0–55.0]	45.0 [42.5–51.0] (*n* = 23)	52.5 [46.2–57.2] (*n* = 30)	0.059

Values were extracted only when transcribed in the free-text record and are not representative of the cohort; they were not used in the mortality models. *p* from the Mann–Whitney U test. CRP, C-reactive protein; IQR, interquartile range; LVEF, left ventricular ejection fraction; WBC, white blood cell count.

**Table 6:** Agreement between automated (rule-based) and manual coding of 22 binary variables, Period 1 (*n* = 144).

Variable	Manual+	Auto+	TP	FP	FN	Acc. %	Sens. %	Spec. %	PPV %	NPV %	Kappa	Grade
Hypertension	109	102	102	0	7	95.1	93.6	100.0	100.0	83.3	0.88	High
Diabetes mellitus	50	43	43	0	7	95.1	86.0	100.0	100.0	93.1	0.89	High
Chronic kidney disease	2	32	1	31	1	77.8	50.0	78.2	3.1	99.1	0.03	Low
Prior stroke	39	40	38	2	1	97.9	97.4	98.1	95.0	99.0	0.95	High
Neoplasm	90	52	52	0	38	73.6	57.8	100.0	100.0	58.7	0.51	Low
Heart failure	58	63	58	5	0	96.5	100.0	94.2	92.1	100.0	0.93	High
COPD/asthma	23	25	23	2	0	98.6	100.0	98.3	92.0	100.0	0.95	High
CDI stated in record	136	135	132	3	4	95.1	97.1	62.5	97.8	55.6	0.56	High
Sepsis/septic shock	57	76	57	19	0	86.8	100.0	78.2	75.0	100.0	0.74	Moderate
GI bleeding	14	17	14	3	0	97.9	100.0	97.7	82.4	100.0	0.89	High
Pneumonia	67	68	67	1	0	99.3	100.0	98.7	98.5	100.0	0.99	High
Urinary tract infection	113	48	45	3	68	50.7	39.8	90.3	93.8	29.2	0.17	Low
Ascites/pleural effusion	91	92	90	2	1	97.9	98.9	96.2	97.8	98.1	0.96	High
CT performed	118	121	118	3	0	97.9	100.0	88.5	97.5	100.0	0.93	High
Endoscopy	25	26	25	1	0	99.3	100.0	99.2	96.2	100.0	0.98	High
Vancomycin	118	118	118	0	0	100.0	100.0	100.0	100.0	100.0	1.00	High
Metronidazole	50	49	49	0	1	99.3	98.0	100.0	100.0	98.9	0.98	High
Probiotics	49	77	48	29	1	79.2	98.0	69.5	62.3	98.5	0.59	Low
Other antibiotics	102	126	102	24	0	83.3	100.0	42.9	81.0	100.0	0.52	Moderate
ICU requirement	74	60	60	0	14	90.3	81.1	100.0	100.0	83.3	0.81	High
Transfer to ID hospital	40	70	38	32	2	76.4	95.0	69.2	54.3	97.3	0.52	Low
In-hospital death	45	45	45	0	0	100.0	100.0	100.0	100.0	100.0	1.00	High

Manual coding is the reference. TN = 144 − TP − FP − FN. Reliability grade: High, accuracy ≥ 90%; moderate, 80–89%; low, <80% (manual verification required). For chronic kidney disease, a targeted audit of the 31 discordant records showed explicit documentation in the narrative for most of them, so the discordance reflects under-coding in the manual reference. Acc., accuracy; Auto+, positives by the algorithm; COPD, chronic obstructive pulmonary disease; FN, false negative; FP, false positive; ICU, intensive care unit; ID, infectious diseases; Manual+, positives by manual coding; NPV, negative predictive value; PPV, positive predictive value; Sens., sensitivity; Spec., specificity; TP, true positive.

## Data Availability

The anonymised patient-level dataset analysed in this study and the analysis script are available as Supplementary Materials. The original clinical records are not publicly available because of privacy and ethical restrictions.

## Supplementary Materials

The following supporting information can be downloaded at: https://www.mdpi.com/article/10.3390/antibiotics15101021/s1, File S1: R script reproducing all tables, figures and additional analyses from Table S1.

## Abbreviations

AUC, area under the receiver operating characteristic curve; CCI, Charlson Comorbidity Index; CDI, *Clostridioides difficile* infection; CI, confidence interval; CNP, national personal identification number; COPD, chronic obstructive pulmonary disease; CT, computed tomography; CLIA, chemiluminescence immunoassay; ESCMID, European Society of Clinical Microbiology and Infectious Diseases; HTA, DZ, BCR, AVC, BPOC, HDS/HDI, ITU and ATI, Romanian clinical abbreviations defined in Section 4.4; ICU, intensive care unit; ID, infectious diseases; IDSA, Infectious Diseases Society of America; IQR, interquartile range; LASSO, least absolute shrinkage and selection operator; LVEF, left ventricular ejection fraction; OR, odds ratio; PCR, polymerase chain reaction; SD, standard deviation; SHEA, Society for Healthcare Epidemiology of America; SOFA, Sequential Organ Failure Assessment; STROBE, Strengthening the Reporting of Observational Studies in Epidemiology; TRIPOD, Transparent Reporting of a multivariable prediction model for Individual Prognosis or Diagnosis.
